# Empirical array quality weights in the analysis of microarray data

**DOI:** 10.1186/1471-2105-7-261

**Published:** 2006-05-19

**Authors:** Matthew E Ritchie, Dileepa Diyagama, Jody Neilson, Ryan van Laar, Alexander Dobrovic, Andrew Holloway, Gordon K Smyth

**Affiliations:** 1 Bioinformatics Division, The Walter and Eliza Hall Institute of Medical Research, 1G Royal Parade, Parkville, Victoria 3050, Australia; 2 lan Potter Foundation Centre for Cancer Genomics and Predictive Medicine, The Peter MacCallum Cancer Centre, St Andrews Place, East Melbourne, Victoria 3002, Australia; 3 Molecular Pathology Research, Department of Pathology, The Peter MacCallum Cancer Centre, St Andrews Place, East Melbourne, Victoria 3002, Australia

## Abstract

**Background:**

Assessment of array quality is an essential step in the analysis of data from microarray experiments. Once detected, less reliable arrays are typically excluded or "filtered" from further analysis to avoid misleading results.

**Results:**

In this article, a graduated approach to array quality is considered based on empirical reproducibility of the gene expression measures from replicate arrays. Weights are assigned to each microarray by fitting a heteroscedastic linear model with shared array variance terms. A novel gene-by-gene update algorithm is used to efficiently estimate the array variances. The inverse variances are used as weights in the linear model analysis to identify differentially expressed genes. The method successfully assigns lower weights to less reproducible arrays from different experiments. Down-weighting the observations from suspect arrays increases the power to detect differential expression. In smaller experiments, this approach outperforms the usual method of filtering the data. The method is available in the limma software package which is implemented in the R software environment.

**Conclusion:**

This method complements existing normalisation and spot quality procedures, and allows poorer quality arrays, which would otherwise be discarded, to be included in an analysis. It is applicable to microarray data from experiments with some level of replication.

## Background

Assessment of data quality is an important component of the analysis pipeline for gene expression microarray experiments [1,2]. Although careful pre-processing and normalisation can ameliorate some problems with microarray data, including background fluorescence, dye effects or spatial artifacts [3], many sources of variation can affect the experimental procedure [4-7] and it is inevitable that variations in data quality will remain. In this article we demonstrate an approach in which variations in data quality are detected and adjusted for as part of the differential expression analysis. The method is widely applicable, easy to use and can have a high payoff.

Quality assessment procedures can be applied at the probe level or at the array level. Probe quality is influenced by local factors on the array such as printing irregularities or spatial artifacts. For spotted microarrays, spot-specific morphology and signal measurements obtained from image analysis software can be used to assign a quality score to each probe on the array [8-11]. Spots with low quality scores are commonly removed from further analysis. An alternative approach is to measure agreement between gene expression values from repeat probes directly and eliminate those spots with inconsistent replicate values [12,13]. For high-density oligonucleotide microarrays with multiple probes per gene, quality measures can be obtained from probe level models (PLMs). Image plots of robust weights or residuals obtained from robust PLMs can highlight artifacts on the array surface [2].

Probe quality assessment is not sufficient because some artifacts only become evident at the array level. Indeed the detection of problems is even more critical at the array level than at the probe level because a single bad array may constitute a sizeable proportion of the data from a microarray experiment. The quality of data from an entire array can be influenced by factors such as sample preparation and day-to-day variability [14]. Sub-standard arrays are typically identified using diagnostic plots of the array data [1,15-17]. The correlation between expression values of repeatedly spotted clones on an array is also used as an array quality measure [18]. Where large data sets are available, a statistical process control approach can identify outlier arrays [19]. In Affymetrix GeneChip experiments, array quality can be assessed using PLM standard errors or from RNA degradation plots [2].

Almost all the methods cited above classify the data as either "good" or "bad", and exclude "bad" probes or arrays from further analysis. In our experience however the "bad" arrays are usually not entirely bad. Very often the lesser quality arrays do contain good information about gene expression but which is embedded in a greater degree of noise than for "good" arrays. In this article, a graduated, quantitative approach is taken to quality at the array level in which poorer quality arrays are included in the analysis but down-weighted.

Quality assessment methods can be divided into those which are "predictive" and those which are "empirical". The operational meaning of quality is that high quality features produce highly reproducible expression values, while low quality features produce values which are more variable and hence less reproducible. Predictive quality assessment methods attempt to predict variability by comparing features such as spot morphology to normative measures. On the other hand, methods which compare duplicate spots within arrays are empirical in that they observe variability.

In this article we extend the empirical approach to multi-array experiments for which we measure the discrepancies between replicate arrays. In order to be as general as possible, we do not limit ourselves to simple replicate experiments, but work with a linear model formulation which allows us to handle experiments of arbitrary complexity including those with factorial or loop designs. The degree of replication in such experiments is reflected in the residual degrees of freedom for computing the residual standard errors. Our method is implemented by way of a heteroscedastic variance model. It is common for statistical models of microarray data to allow each probe to have its own individual variance. Our heteroscedastic model allows the variance to depend on the array as well as on the probe. The array variance factors then enter into the subsequent analysis as inverse array quality weights. Importantly, our method not only detects variations in data quality but adjusts for this as part of the analysis.

Our approach can be combined with predictive quality assessment methods and is an effective complement to them. Predictive methods can be used to filter spots or to provide quantitative prior spot weights which are incorporated into the linear model analysis. However the causes of poor quality data cannot always be clearly identified. The empirical array weight method described here estimates and accommodates any variation in quality which remains after the spot quality weights have been taken into account, i.e., after prediction has achieved as much as it can. Our approach is particularly effective when arrays vary in quality but the problems cannot be isolated to particular regions or particular probes on the offending arrays.

The presence of array-level parameters in our heteroscedastic model means that the statistical analysis can no longer be undertaken in a purely gene-wise manner. A naive approach to fitting the model would be computationally expensive. We propose two computationally efficient algorithms for estimating the model by the well-recognised statistical criterion of residual maximum likelihood (REML). These algorithms view the microarray data as many small data sets, one for each probe, with a small number of shared parameters corresponding to the array variance factors. An innovative gene-by-gene update procedure is proposed for particularly fast approximate REML estimation.

The array weight method developed here can be applied to any microarray experiment with array-level replication, including experiments using high-density oligonucleotide arrays, but our experience is mainly with experiments using spotted microarrays. High density arrays allow the additional possibility of measuring reproducibility for multiple probes for each gene rather than relying on gene or probe-set summaries [2]. A full treatment of empirical array quality for these platforms is therefore likely to involve an analysis of reproducibility at both the probe level and probe-set level, a further development which is not investigated in this article.

In this paper, the linear model approach to microarray data analysis is reviewed and the heteroscedastic model which includes array weights is introduced. Next, the experimental and simulated data sets used in this study are explained and results for these data are presented. The computational algorithms for fitting the heteroscedastic model are then described, followed by discussion and conclusions. Supplementary materials including data, R scripts and additional plots are available [20].

## Linear models for microarray data

Linear models provide a convenient means to measure and test for differential expression in microarray experiments involving many different RNA sources [21,22]. The linear model approach allows a unified treatment of a wide variety of microarray experiments, including dye-swaps, common reference experiments, factorial experiments and loop or saturated designs, with little more complication than simple replicated experiments. Although the statement of the linear model, given below, requires some mathematical notation, the application of the methods we describe is in practice very simple using available software. Consider a microarray experiment with expression values *y*_*gj *_for genes *g *= 1, ..., *G *and arrays *j *= 1, ..., *J*. The expression values could be log-ratios from two-colour microarrays or summarised log-intensity values from a single-channel technology such as Affymetrix GeneChips. We assume that the expression values have been appropriately pre-processed, background corrected and normalised. The term *gene *is used here in a general way to include any ESTs or control probes that might be on the arrays. Assume that the systematic expression effects for each gene can be described by a linear model

*E*(**y**_*g*_) = *X**β ***_*g *_    (1)

where **y**_*g *_= (*y*_*g*1_, ..., *y*_*gJ*_)^*T *^is the vector of expression values for gene *g, X *is a known design matrix with full column rank *K*, and ***β***_*g *_= (*β*_*g*1_, ..., *β *_*gK*_)^*T *^is a gene-specific vector of regression coefficients. The design matrix will depend upon the experimental design and choice of parameterisation and the regression coefficients represent log-fold changes between RNA sources in the experiment [22,23]. For example, consider a two-colour microarray experiment with three replicate arrays comparing RNA sources *A *and *B*. The individual log-ratios *y*_*gj *_= log_2_(*R*_*gj*_*/G*_*gj*_), where *R*_*gj *_and *G*_*gj *_are the Cy5 and Cy3 intensities, measure differences in gene expression between the two samples. For a simple replicated experiment with sample *B *always labelled Cy5, the design matrix would be a column of ones, and the coefficient *β*_*g *_would represent the log-fold-change for gene *g *in sample *B *over *A*. Replicated experiments with dye-swaps would be the same except that minus ones would indicate the dye-swap arrays. Consider another example where samples *A *and *B *are compared through a common reference sample. If there are two arrays for each sample and the common reference is always Cy3, then the design matrix would be

X=(10101111).
 MathType@MTEF@5@5@+=feaafiart1ev1aaatCvAUfKttLearuWrP9MDH5MBPbIqV92AaeXatLxBI9gBaebbnrfifHhDYfgasaacH8akY=wiFfYdH8Gipec8Eeeu0xXdbba9frFj0=OqFfea0dXdd9vqai=hGuQ8kuc9pgc9s8qqaq=dirpe0xb9q8qiLsFr0=vr0=vr0dc8meaabaqaciaacaGaaeqabaqabeGadaaakeaacqWGybawcqGH9aqpdaqadaqaauaabaaaeiaaaaqaaiabigdaXaqaaiabicdaWaqaaiabigdaXaqaaiabicdaWaqaaiabigdaXaqaaiabigdaXaqaaiabigdaXaqaaiabigdaXaaaaiaawIcacaGLPaaacqGGUaGlaaa@38E8@

Here the first coefficient *β*_*g*1 _estimates the log-fold-change between *A *and the common reference while the second coefficient *β*_*g*2 _estimates the comparison of interest between *B *and *A*. The design matrix can be expanded indefinitely to represent experiments of arbitrary complexity.

The linear model also assumes

var(*y*_*gj*_) = σg2
 MathType@MTEF@5@5@+=feaafiart1ev1aaatCvAUfKttLearuWrP9MDH5MBPbIqV92AaeXatLxBI9gBaebbnrfifHhDYfgasaacH8akY=wiFfYdH8Gipec8Eeeu0xXdbba9frFj0=OqFfea0dXdd9vqai=hGuQ8kuc9pgc9s8qqaq=dirpe0xb9q8qiLsFr0=vr0=vr0dc8meaabaqaciaacaGaaeqabaqabeGadaaakeaaiiGacqWFdpWCdaqhaaWcbaGaem4zaCgabaGaeGOmaidaaaaa@30EC@/*w*_*gj *_    (2)

where *w*_*gj *_is a prior spot quality weight and σg2
 MathType@MTEF@5@5@+=feaafiart1ev1aaatCvAUfKttLearuWrP9MDH5MBPbIqV92AaeXatLxBI9gBaebbnrfifHhDYfgasaacH8akY=wiFfYdH8Gipec8Eeeu0xXdbba9frFj0=OqFfea0dXdd9vqai=hGuQ8kuc9pgc9s8qqaq=dirpe0xb9q8qiLsFr0=vr0=vr0dc8meaabaqaciaacaGaaeqabaqabeGadaaakeaaiiGacqWFdpWCdaqhaaWcbaGaem4zaCgabaGaeGOmaidaaaaa@30EC@ is the unknown gene-specific variance factor. The spot quality weights will usually have arisen from a predictive spot quality assessment step, with large weights representing good quality spots and low weights representing poor quality spots. To avoid unnecessary complications we will assume throughout that all the *y*_*gj *_are observed and that all the spot weights are strictly positive, *w*_*gj *_> 0. In practice, the methods developed in this article can be modified to accommodate missing *y-*values or zero weights, but this complicates the presentation somewhat and will be omitted.

For simplicity we will assume that the *y*_*gj *_are normally distributed and that expression values from different arrays are independent. The weighted least squares estimator of **β**_*g *_is

β^g=(XTΣg−1X)−1XTΣg−1yg     (3)
 MathType@MTEF@5@5@+=feaafiart1ev1aaatCvAUfKttLearuWrP9MDH5MBPbIqV92AaeXatLxBI9gBaebbnrfifHhDYfgasaacH8akY=wiFfYdH8Gipec8Eeeu0xXdbba9frFj0=OqFfea0dXdd9vqai=hGuQ8kuc9pgc9s8qqaq=dirpe0xb9q8qiLsFr0=vr0=vr0dc8meaabaqaciaacaGaaeqabaqabeGadaaakeaaiiWacuWFYoGygaqcamaaBaaaleaacqWGNbWzaeqaaOGaeyypa0JaeiikaGIaemiwaG1aaWbaaSqabeaacqWGubavaaGccqqHJoWudaqhaaWcbaGaem4zaCgabaGaeyOeI0IaeGymaedaaOGaemiwaGLaeiykaKYaaWbaaSqabeaacqGHsislcqaIXaqmaaGccqWGybawdaahaaWcbeqaaiabdsfaubaakiabfo6atnaaDaaaleaacqWGNbWzaeaacqGHsislcqaIXaqmaaacbeGccqGF5bqEdaWgaaWcbaacbaGae03zaCgabeaakiaaxMaacaWLjaWaaeWaaeaacqaIZaWmaiaawIcacaGLPaaaaaa@4BE8@

where Σ_*g *_= diag(*w*_*g*1_, ..., *w*_*gJ*_) is the diagonal matrix of prior weights. The *t*-statistic for testing any particular *β*_*gk *_equal to zero is

tgk=β^gksgcgk
 MathType@MTEF@5@5@+=feaafiart1ev1aaatCvAUfKttLearuWrP9MDH5MBPbIqV92AaeXatLxBI9gBaebbnrfifHhDYfgasaacH8akY=wiFfYdH8Gipec8Eeeu0xXdbba9frFj0=OqFfea0dXdd9vqai=hGuQ8kuc9pgc9s8qqaq=dirpe0xb9q8qiLsFr0=vr0=vr0dc8meaabaqaciaacaGaaeqabaqabeGadaaakeaacqWG0baDdaWgaaWcbaGaem4zaCMaem4AaSgabeaakiabg2da9maalaaabaacciGaf8NSdiMbaKaadaWgaaWcbaGaem4zaCMaem4AaSgabeaaaOqaaiabdohaZnaaBaaaleaacqWGNbWzaeqaaOWaaOaaaeaacqWGJbWydaWgaaWcbaGaem4zaCMaem4AaSgabeaaaeqaaaaaaaa@3E00@

where sg2
 MathType@MTEF@5@5@+=feaafiart1ev1aaatCvAUfKttLearuWrP9MDH5MBPbIqV92AaeXatLxBI9gBaebbnrfifHhDYfgasaacH8akY=wiFfYdH8Gipec8Eeeu0xXdbba9frFj0=OqFfea0dXdd9vqai=hGuQ8kuc9pgc9s8qqaq=dirpe0xb9q8qiLsFr0=vr0=vr0dc8meaabaqaciaacaGaaeqabaqabeGadaaakeaacqWGZbWCdaqhaaWcbaGaem4zaCgabaGaeGOmaidaaaaa@3091@ is the residual mean square from weighted regression and *c*_*gk *_is the *k*th diagonal element of (XTΣg−1X)−1
 MathType@MTEF@5@5@+=feaafiart1ev1aaatCvAUfKttLearuWrP9MDH5MBPbIqV92AaeXatLxBI9gBaebbnrfifHhDYfgasaacH8akY=wiFfYdH8Gipec8Eeeu0xXdbba9frFj0=OqFfea0dXdd9vqai=hGuQ8kuc9pgc9s8qqaq=dirpe0xb9q8qiLsFr0=vr0=vr0dc8meaabaqaciaacaGaaeqabaqabeGadaaakeaacqGGOaakcqWGybawdaahaaWcbeqaaiabdsfaubaakiabfo6atnaaDaaaleaacqWGNbWzaeaacqGHsislcqaIXaqmaaGccqWGybawcqGGPaqkdaahaaWcbeqaaiabgkHiTiabigdaXaaaaaa@3931@.

It is important to appreciate that the spot weights *w*_*gj *_act in a relative fashion for each gene. The *t*-statistic *t*_*gk *_and its associated *p-*value would be unchanged if all the *w*_*gj *_for a given *g *were scaled up or down by a constant factor. Hence it is only the relative sizes of the *w*_*gj *_across arrays *j *for any given *g *which are important.

The *t*-statistic has *J *- *K *degrees of freedom. In microarray analyses with a small to moderate number of arrays, for which *J *- *K *is small, it is usually beneficial to replace sg2
 MathType@MTEF@5@5@+=feaafiart1ev1aaatCvAUfKttLearuWrP9MDH5MBPbIqV92AaeXatLxBI9gBaebbnrfifHhDYfgasaacH8akY=wiFfYdH8Gipec8Eeeu0xXdbba9frFj0=OqFfea0dXdd9vqai=hGuQ8kuc9pgc9s8qqaq=dirpe0xb9q8qiLsFr0=vr0=vr0dc8meaabaqaciaacaGaaeqabaqabeGadaaakeaacqWGZbWCdaqhaaWcbaGaem4zaCgabaGaeGOmaidaaaaa@3091@ with a variance estimator which is shrunk or moderated across genes to obtain moderated *t*-statistics [22]. Genes can then be selected for differential expression based on large moderated *t*-statistics or small *p-*values.

## A heteroscedastic model for probes and arrays

In this article we allow the unknown variance factors to depend on the array as well as on the gene,

var(*y*_*gj*_) = σgj2
 MathType@MTEF@5@5@+=feaafiart1ev1aaatCvAUfKttLearuWrP9MDH5MBPbIqV92AaeXatLxBI9gBaebbnrfifHhDYfgasaacH8akY=wiFfYdH8Gipec8Eeeu0xXdbba9frFj0=OqFfea0dXdd9vqai=hGuQ8kuc9pgc9s8qqaq=dirpe0xb9q8qiLsFr0=vr0=vr0dc8meaabaqaciaacaGaaeqabaqabeGadaaakeaaiiGacqWFdpWCdaqhaaWcbaGaem4zaCMaemOAaOgabaGaeGOmaidaaaaa@3249@/*w*_*gj*_.     (4)

We need a model for the variance factors σgj2
 MathType@MTEF@5@5@+=feaafiart1ev1aaatCvAUfKttLearuWrP9MDH5MBPbIqV92AaeXatLxBI9gBaebbnrfifHhDYfgasaacH8akY=wiFfYdH8Gipec8Eeeu0xXdbba9frFj0=OqFfea0dXdd9vqai=hGuQ8kuc9pgc9s8qqaq=dirpe0xb9q8qiLsFr0=vr0=vr0dc8meaabaqaciaacaGaaeqabaqabeGadaaakeaaiiGacqWFdpWCdaqhaaWcbaGaem4zaCMaemOAaOgabaGaeGOmaidaaaaa@3249@ which reflects the fact that the genes differ in variability and also that the arrays in the experiment may differ in quality in a way which increases or decreases the variability of all or most of the probes on a particular array. The simplest model which does this is the additive log-linear model

log σgj2
 MathType@MTEF@5@5@+=feaafiart1ev1aaatCvAUfKttLearuWrP9MDH5MBPbIqV92AaeXatLxBI9gBaebbnrfifHhDYfgasaacH8akY=wiFfYdH8Gipec8Eeeu0xXdbba9frFj0=OqFfea0dXdd9vqai=hGuQ8kuc9pgc9s8qqaq=dirpe0xb9q8qiLsFr0=vr0=vr0dc8meaabaqaciaacaGaaeqabaqabeGadaaakeaaiiGacqWFdpWCdaqhaaWcbaGaem4zaCMaemOAaOgabaGaeGOmaidaaaaa@3249@ = *δ*_*g *_+ *γ*_*j *_    (5)

[24,25]. We impose the constraint ∑j=1Jγj=0
 MathType@MTEF@5@5@+=feaafiart1ev1aaatCvAUfKttLearuWrP9MDH5MBPbIqV92AaeXatLxBI9gBaebbnrfifHhDYfgasaacH8akY=wiFfYdH8Gipec8Eeeu0xXdbba9frFj0=OqFfea0dXdd9vqai=hGuQ8kuc9pgc9s8qqaq=dirpe0xb9q8qiLsFr0=vr0=vr0dc8meaabaqaciaacaGaaeqabaqabeGadaaakeaadaaeWaqaaiabeo7aNnaaBaaaleaacqWGQbGAaeqaaaqaaiabdQgaQjabg2da9iabigdaXaqaaiabdQeakbqdcqGHris5aOGaeyypa0JaeGimaadaaa@3841@ so that the σg2
 MathType@MTEF@5@5@+=feaafiart1ev1aaatCvAUfKttLearuWrP9MDH5MBPbIqV92AaeXatLxBI9gBaebbnrfifHhDYfgasaacH8akY=wiFfYdH8Gipec8Eeeu0xXdbba9frFj0=OqFfea0dXdd9vqai=hGuQ8kuc9pgc9s8qqaq=dirpe0xb9q8qiLsFr0=vr0=vr0dc8meaabaqaciaacaGaaeqabaqabeGadaaakeaaiiGacqWFdpWCdaqhaaWcbaGaem4zaCgabaGaeGOmaidaaaaa@30EC@ = exp *δ*_*g *_represent the gene-wise variance factors while the *γ*_*j *_represent the relative variability of each array. Array *j *will have *γ*_*j *_< 0 or *γ*_*j *_> 0 depending on whether it is relatively better or poorer quality than the average. For instance, an array with exp *γ*_*j *_= 2 is twice as variable as a typical array and will be given half weight in an analysis. Note that the variances are assumed to depend multiplicatively on array quality. This is more appropriate than, say, an additive model of gene and array variances because it preserves relativities between the gene-wise precisions as array quality varies. The log-linear variance model also has substantial numerical and inferential advantages over other variance models in that positivity for the variances is ensured for any values of the *δ*_*g *_and *γ*_*j *_parameters.

The fact that all the genes contribute to the estimation of the *γ*_*j *_means that, once estimated, the array weights can be taken to be fixed quantities when analysing each individual gene. The array weights *v*_*j *_= 1/exp γ^j
 MathType@MTEF@5@5@+=feaafiart1ev1aaatCvAUfKttLearuWrP9MDH5MBPbIqV92AaeXatLxBI9gBaebbnrfifHhDYfgasaacH8akY=wiFfYdH8Gipec8Eeeu0xXdbba9frFj0=OqFfea0dXdd9vqai=hGuQ8kuc9pgc9s8qqaq=dirpe0xb9q8qiLsFr0=vr0=vr0dc8meaabaqaciaacaGaaeqabaqabeGadaaakeaaiiGacuWFZoWzgaqcamaaBaaaleaacqWGQbGAaeqaaaaa@2FF3@ can be incorporated into a differential expression analysis simply by combining them with the prior weights into modified weights wgj∗
 MathType@MTEF@5@5@+=feaafiart1ev1aaatCvAUfKttLearuWrP9MDH5MBPbIqV92AaeXatLxBI9gBaebbnrfifHhDYfgasaacH8akY=wiFfYdH8Gipec8Eeeu0xXdbba9frFj0=OqFfea0dXdd9vqai=hGuQ8kuc9pgc9s8qqaq=dirpe0xb9q8qiLsFr0=vr0=vr0dc8meaabaqaciaacaGaaeqabaqabeGadaaakeaacqWG3bWDdaqhaaWcbaGaem4zaCMaemOAaOgabaGaey4fIOcaaaaa@31F3@ = *w*_*gj*_*v*_*j*_. The weighted least squares calculations described in the previous section (Equation 3) can then be conducted with wgj∗
 MathType@MTEF@5@5@+=feaafiart1ev1aaatCvAUfKttLearuWrP9MDH5MBPbIqV92AaeXatLxBI9gBaebbnrfifHhDYfgasaacH8akY=wiFfYdH8Gipec8Eeeu0xXdbba9frFj0=OqFfea0dXdd9vqai=hGuQ8kuc9pgc9s8qqaq=dirpe0xb9q8qiLsFr0=vr0=vr0dc8meaabaqaciaacaGaaeqabaqabeGadaaakeaacqWG3bWDdaqhaaWcbaGaem4zaCMaemOAaOgabaGaey4fIOcaaaaa@31F3@ replacing *w*_*gj *_throughout. The use of appropriate array weights will produce more precise estimates of the gene expression coefficients and improve power to detect differentially expressed genes.

Note that, although the scaling of the array weights is in principle arbitrary, our convention that ∑j=1Jγj=0
 MathType@MTEF@5@5@+=feaafiart1ev1aaatCvAUfKttLearuWrP9MDH5MBPbIqV92AaeXatLxBI9gBaebbnrfifHhDYfgasaacH8akY=wiFfYdH8Gipec8Eeeu0xXdbba9frFj0=OqFfea0dXdd9vqai=hGuQ8kuc9pgc9s8qqaq=dirpe0xb9q8qiLsFr0=vr0=vr0dc8meaabaqaciaacaGaaeqabaqabeGadaaakeaadaaeWaqaaiabeo7aNnaaBaaaleaacqWGQbGAaeqaaaqaaiabdQgaQjabg2da9iabigdaXaqaaiabdQeakbqdcqGHris5aOGaeyypa0JaeGimaadaaa@3841@ means we always choose the array weights *v*_*j *_to have geometric mean equal to one.

### Data

The use of array quality weights will be demonstrated on both real and simulated data sets. The first data set was acquired as a quality control step in the array fabrication process at the Peter MacCallum Cancer Centre, Melbourne. This data set contains 100 microarrays representing 4–6 arrays taken from the beginning, middle and end of 22 different print batches. The arrays were printed with a human 10.5 K cDNA library and six copies of the Lucidea Microarray ScoreCard (LMS) set of control probes [26]. Each array was hybridised with Cy3 labelled mRNA from the MCF7 breast cancer cell line and Cy5 labelled mRNA from the Jurkat T-cell leukemia cell line. Test and reference LMS spike-in mixes were added to the mRNA samples prior to labelling to produce predictable fold changes for the control spots (Table 1). The ratio control spots should show three-fold or ten-fold changes while the dynamic range spots should not be differentially expressed. The array images were analysed using Spot 2.0 [27] and the intensities were background corrected by subtracting morphological (*morph*) background values. The *morph *background treatment ensures that all intensities remain positive after background correction, and damps down the variability of the log-ratios for low intensity spots [28]. This eliminates the need for intensity-based filtering of spots in the subsequent analysis. We use this data for two purposes. Firstly, log-ratios were print-tip loess normalised [29]. Standardised residuals, (*y*_*gj *_- y¯g
 MathType@MTEF@5@5@+=feaafiart1ev1aaatCvAUfKttLearuWrP9MDH5MBPbIqV92AaeXatLxBI9gBaebbnrfifHhDYfgasaacH8akY=wiFfYdH8Gipec8Eeeu0xXdbba9frFj0=OqFfea0dXdd9vqai=hGuQ8kuc9pgc9s8qqaq=dirpe0xb9q8qiLsFr0=vr0=vr0dc8meaabaqaciaacaGaaeqabaqabeGadaaakeaaieGacuWF5bqEgaqeamaaBaaaleaacqWFNbWzaeqaaaaa@2FC5@)/*s*_*g*_, were computed where *y*_*gj *_are the normalised log-ratios and y¯g
 MathType@MTEF@5@5@+=feaafiart1ev1aaatCvAUfKttLearuWrP9MDH5MBPbIqV92AaeXatLxBI9gBaebbnrfifHhDYfgasaacH8akY=wiFfYdH8Gipec8Eeeu0xXdbba9frFj0=OqFfea0dXdd9vqai=hGuQ8kuc9pgc9s8qqaq=dirpe0xb9q8qiLsFr0=vr0=vr0dc8meaabaqaciaacaGaaeqabaqabeGadaaakeaaieGacuWF5bqEgaqeamaaBaaaleaacqWFNbWzaeqaaaaa@2FC5@ and *s*_*g *_are the probe-wise means and standard deviations. Standardised residuals from the 75% most highly expressed probes in the 10.5 K cDNA library were used as a population of non-normal deviates for generating simulated data sets. The other analysis of this data uses only the 120 LMS control spots on each array. Log-ratios from these control spots were global loess normalised [29], using a relatively wide span of 0.7 because of the relatively small number of spots used. The resulting data will be referred to as the QC LMS data set in the remainder of the article.

**Figure 1:**
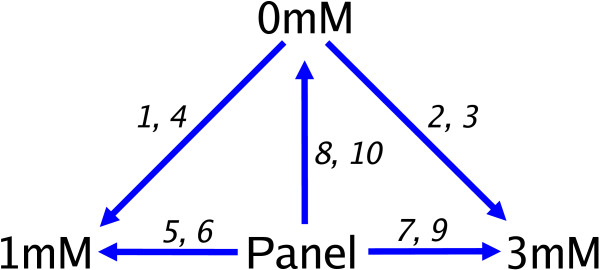
**Design of the METH experiment**. The METH experiment compared 3 mRNA sources of interest (0 mM, 1 mM and 3 mM) directly on the first 4 arrays and indirectly via a Panel reference on a further 6 arrays. The arrays are numbered from 1 to 10 in the order they were hybridised. Each arrow indicates a direct comparison made within an array, and points from the Cy3 labelled sample towards the Cy5 labelled sample.

The second data set arose from a study aimed at identifying novel methylated markers in myeloid malignancy using the leukemia cell line KG1a. Microarrays were printed with the same cDNA library and controls as the first data set. A known inhibitor of DNA methylation, 5-azacytidine, was added to KG1a cells in varying doses (0 mM, 1 mM and 3 mM). Both direct and indirect comparisons between the 1 mM and 3 mM treatments and the 0 mM treatment were made on a total of 10 arrays (Figure 1). The panel reference RNA consisted of a pool of RNA from 11 cancer cell lines. The arrays were scanned on a GenePix 4000B scanner and image analysed using GenePix Pro 4.0. The intensities were background corrected using the model-based 'normexp' method with an offset of 50 [30]. Again, this background correction method avoids negative intensities and the need for intensity-based filtering. Log-ratios were print-tip loess normalised [29]. This data set will be referred to as the METH experiment.

### Simulations

For the simulation studies, normal and non-normal expression values (*y*_*gj*_) from replicate arrays were generated with *G *= 10000 genes and *J *= 3 and 5 arrays in six different scenarios. For each simulation, different array variances (exp *γ*_*j*_) were assumed, and the gene-specific variances (exp *δ*_*g*_) were sampled from the estimates (sg2
 MathType@MTEF@5@5@+=feaafiart1ev1aaatCvAUfKttLearuWrP9MDH5MBPbIqV92AaeXatLxBI9gBaebbnrfifHhDYfgasaacH8akY=wiFfYdH8Gipec8Eeeu0xXdbba9frFj0=OqFfea0dXdd9vqai=hGuQ8kuc9pgc9s8qqaq=dirpe0xb9q8qiLsFr0=vr0=vr0dc8meaabaqaciaacaGaaeqabaqabeGadaaakeaacqWGZbWCdaqhaaWcbaGaem4zaCgabaGaeGOmaidaaaaa@3091@) obtained from the QC data set. Non-normal deviates were sampled from the standardised residuals of the QC data set. These deviates are considerably more heavy-tailed than normal. In each data set, 5% (500) of genes were simulated to be differentially expressed at either 2-fold (250) or 3-fold (250), while the remaining 95% were simulated to have mean zero.

For the simulations with 3 arrays, the expression values for the third array were generated to be twice as variable as those from the first two arrays in simulation 1 (i.e., *v*_1 _= *v*_2 _= 2*v*_3_), ten times as variable as the first two arrays in simulation 2 (i.e., *v*_1 _= *v*_2 _= 10*v*_3_) or five times more variable on the second array and ten times more variable on the third array relative to the first in simulation 3 (i.e., *v*_1 _= 5*v*_2 _= 10*v*_3_).

Simulations with 5 arrays were generated to have at least two more variable arrays. In simulation 4, expression values on the fourth and fifth arrays were simulated to be two times and four times more variable than those on the first three arrays (i.e., *v*_1 _= *v*_2 _= *v*_3 _= 2*v*_4 _= 4*v*_5_). In simulation 5, expression values from the fourth and fifth arrays were five and ten times more variable than those on the first three arrays (i.e., *v*_1 _= *v*_2 _= *v*_3 _= 5*v*_4 _= 10*v*_5_). For simulation 6, the expression values were two times, four times, six times and ten times more variable on arrays two to five respectively relative to the first array (i.e., *v*_1 _= 2*v*_2 _= 4*v*_3 _= 6*v*_4 _= 10*v*_5_).

The six different scenarios and the true array weights in each case are listed in the first two columns of Table 2. Recall that only the relative sizes of the array weights are relevant so, by the convention described earlier (∑j=1Jγj=0
 MathType@MTEF@5@5@+=feaafiart1ev1aaatCvAUfKttLearuWrP9MDH5MBPbIqV92AaeXatLxBI9gBaebbnrfifHhDYfgasaacH8akY=wiFfYdH8Gipec8Eeeu0xXdbba9frFj0=OqFfea0dXdd9vqai=hGuQ8kuc9pgc9s8qqaq=dirpe0xb9q8qiLsFr0=vr0=vr0dc8meaabaqaciaacaGaaeqabaqabeGadaaakeaadaaeWaqaaiabeo7aNnaaBaaaleaacqWGQbGAaeqaaaqaaiabdQgaQjabg2da9iabigdaXaqaaiabdQeakbqdcqGHris5aOGaeyypa0JaeGimaadaaa@3841@), we always scale the array weights so that they have geometric mean equal to one.

## Results

### Simulations

First we demonstrate the ability of the algorithms to return the correct array weights for simulated data sets where the true array variances are known. For each of the six simulation scenarios described in the previous section, 1000 independent data sets were generated and the variance model (Equation 5) was fitted to each. This was carried out for both normal and non-normal data. For each data set, estimates were obtained using the full REML algorithm and the approximate gene-by-gene update algorithm (see Methods section). Table 2 shows the means and standard deviations of the estimated array weights ^*v*^*j*. The full algorithm is shown to assign weights almost exactly consistent with the predicted values. The gene-by-gene update method returns array weights which are slightly less extreme, i.e., slightly flattened towards equal weights, although still broadly accurate. The gene-by-gene estimates are also somewhat more variable than those for full REML, a consequence of the fact that the REML estimators are theoretically optimal. All the standard deviations are small enough however that the variability is negligible, even for the approximate algorithm. The results are virtually unchanged whether the data is normal or non-normal. Although the accuracy of the full REML algorithm is impressive here, it is important to appreciate that very precise estimates of the array variances are not required for a weighted analysis to be effective, so that the gene-by-gene algorithm may be adequate in practice.

Note also that the REML algorithms are invariant with respect to the gene-wise means or standard deviations, so the results given in Table 2 remain the same regardless of how the gene specific means or standard deviations are generated.

**Figure 2:**
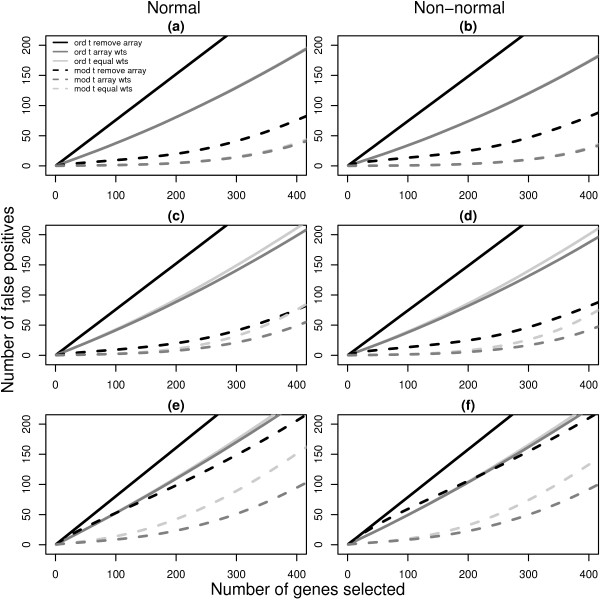
**Number of false discoveries from simulated data sets with 3 arrays**. For each 3 array simulation in Table 2 (1, 2 and 3), the average false discovery rates calculated using ordinary *t*-statistics (solid lines) or moderated *t*-statistics (dashed lines) are given. Panels (a), (c) and (e) show the results for normal data under simulations 1, 2 and 3 respectively while panels (b), (d) and (f) display the corresponding results for non-normal data. Black lines plot the false discovery rates when the most unreliable array is removed from the analysis. Light gray lines are the results obtained using equal weights and dark gray lines show the false discovery rates recovered with array weights. Each line is the average of 1000 simulated data sets. In nearly all cases, the use of array weights in the analysis gives fewer false positives than the other methods. Simulation 1 is the exception, with equal weighting and array weighting producing similar false discovery rates (overlapping curves) for both normal (a) and non-normal (b) data.

**Figure 3:**
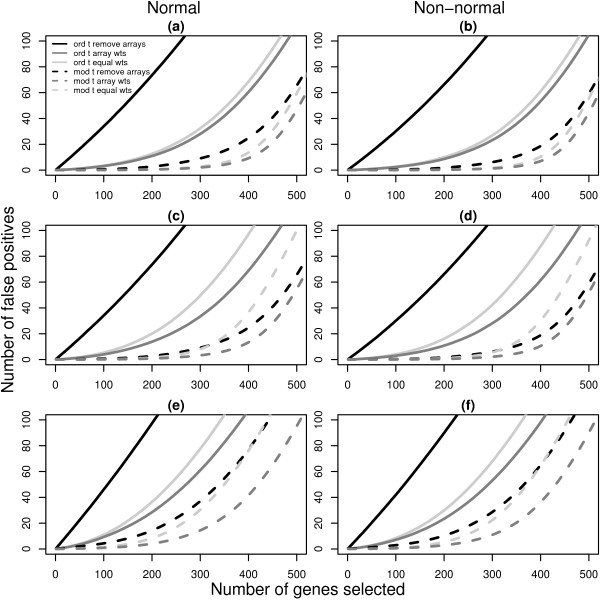
**Number of false discoveries from simulated data sets with 5 arrays**. For each 5 array simulation in Table 2 (4, 5 and 6), the average false discovery rates calculated using ordinary *t*-statistics (solid lines) or moderated *t*-statistics (dashed lines) are given. Panels (a), (c) and (e) show the results for normal data under simulations 4, 5 and 6 respectively while panels (b), (d) and (f) display the corresponding results for non-normal data. Black lines plot the false discovery rates when the two most unreliable arrays are removed from the analysis. Light gray lines are the results obtained using equal weights and dark gray lines show the false discovery rates recovered with array weights. Each line is the average of 1000 simulated data sets. In all cases, the use of array weights in the analysis gives fewer false positives than the other methods.

Next we turn to the detection of differential expression and false discovery rates. For each simulated data set, differentially expressed genes were selected using ordinary *t*-statistics and using the empirical Bayes moderated *t*-statistics implemented in the limma software package [30]. These differential expression measures were used to compare three different array weighting schemes. We considered that an experimenter might choose (i) to use all the arrays equally in the analysis (equal weights), (ii) to use the array weights estimated by the REML algorithm, or (iii) to remove the worst one or two arrays from the analysis entirely (filtering). False discovery rates were calculated to compare the three weighting schemes. Figure 2 shows the average number of false discoveries plotted against the number of genes selected using ordinary *t*-statistics (solid lines) or moderated *t*-statistics (dashed lines) for the 3 array simulations listed in Table 2. Each line represents the average of 1000 simulations. Panels (a), (c) and (e) show the normal results for simulation 1, 2 and 3 respectively, while panels (b), (d) and (f) give the corresponding results for non-normal data. The same layout is used in Figure 3 for the 5 array simulations.

The black lines show the results obtained after removing the most variable array from simulations 1, 2 and 3 (Figure 2), or after removing the two most variable arrays in simulations 4, 5 and 6 (Figure 3). The light gray lines show the number of false positives obtained using equal weights and the dark gray lines indicate the false discovery rates when array weights from the full REML algorithm are used.

The first striking feature of Figures 2 and 3 is that the moderated *t*-statistics easily outperform the ordinary *t*-statistics regardless of the simulation assumptions, consistent with findings in other studies [22,31]. The second feature is that the use of array weights always gives the lowest false discovery rate of the three weighting schemes, regardless of which *t*-statistic is used. Array weighting outperforms both equal weighting and array filtering in all cases, although in simulation 1 equal weighting is nearly as good (the dark gray and light gray lines overlap in Figure 2, panels a and b). It is interesting that the strategy most commonly proposed in the literature, that of array-filtering, is generally the worst performer across the scenarios, except in simulation 5 with moderated *t*-statistics, when equal weighting is worst. The use of array-filtering with ordinary *t*-statistics is very poor indeed. This is despite the fact that the simulation results make array-filtering appear somewhat better than it could be in practice. This is because we always removed the one or two arrays which were known to be the most variable, whereas in real data situations the true status of each array is uncertain and must be inferred using diagnostic plots or other methods. The results in Figures 2 and 3 are for the full REML algorithm, however the results are virtually identical when the approximate gene-by-gene update algorithm is used instead [20]. This shows that the differences in estimated weights between the full and approximate REML algorithms observed in Table 2 are relatively unimportant from the point of view of evaluating differential expression.

### QC LMS Data

**Figure 4:**
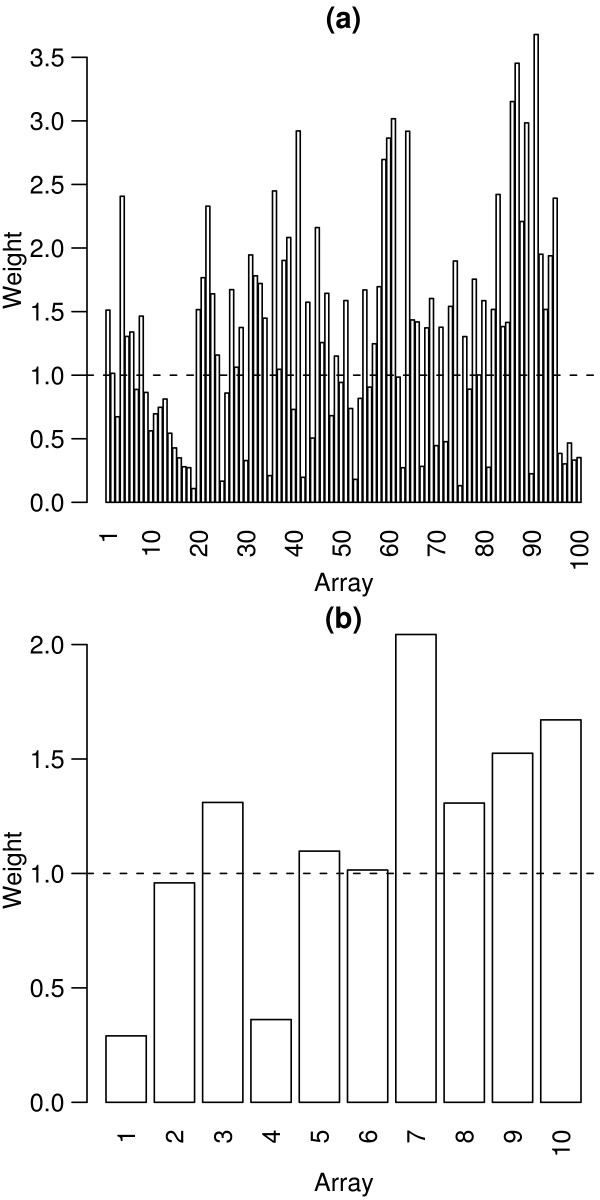
**Array weights for the QC LMS and METH data sets**. Array weights (*v*_*j*_) calculated for the QC LMS data set (a) and the METH experiment (b). The arrays are ordered by time of hybridisation, and the dashed lines show the unit weight. In each experiment, there are arrays which receive higher and lower relative weights, corresponding to arrays which are more or less reproducible.

**Figure 5:**
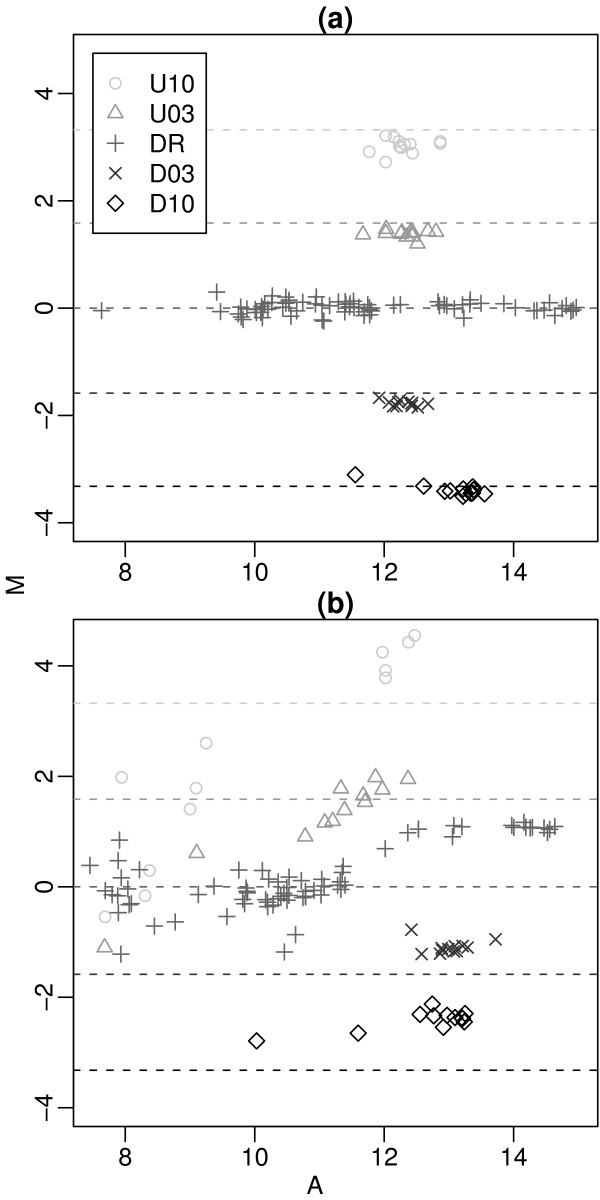
**MA-plots of QC LMS controls for two arrays**. MA-plots for arrays 91 (a) and 19 (b) which were assigned the highest and lowest quality weights respectively. Here *M *= log_2_(*R/G*) is the spot log-ratio and *A *= (log_2_*G *+ log_2_*R*)/2 is the spot log-intensity. Dashed lines show the theoretical *M *values from Table 1. The controls from array 91 consistently recover the true spike-in log-ratios and are assigned high weight (*v*_91 _= 3.68), whereas the log-ratios from array 19 are considerably more variable, resulting in a very low weight (*v*_91 _= 0.11).

The QC LMS data set provides an example of real data where we know the differential expression status of each spot. This example has the structure of a simple replicated experiment. The very large number of replicates allows us to assess accurately the effect of array weights. The variance model (Equation 5) was fitted to the log-ratios for the 120 LMS control spots across the 100 arrays. The array weights, *v*_*j *_= 1/exp γ^j
 MathType@MTEF@5@5@+=feaafiart1ev1aaatCvAUfKttLearuWrP9MDH5MBPbIqV92AaeXatLxBI9gBaebbnrfifHhDYfgasaacH8akY=wiFfYdH8Gipec8Eeeu0xXdbba9frFj0=OqFfea0dXdd9vqai=hGuQ8kuc9pgc9s8qqaq=dirpe0xb9q8qiLsFr0=vr0=vr0dc8meaabaqaciaacaGaaeqabaqabeGadaaakeaaiiGacuWFZoWzgaqcamaaBaaaleaacqWGQbGAaeqaaaaa@2FF3@, are shown in Figure 4(a). The weights vary from a minimum of 0.11 for array 19 to a maximum of 3.68 for array 91. The least squares estimate of the log-fold change between the two RNA sources for each gene is the weighted mean of the individual array log-ratios with these weights. Inspection of MA-plots shows that arrays with lower estimated weights do indeed appear to return the theoretical fold changes more poorly than arrays with higher weights (Figure 5).

**Figure 6:**
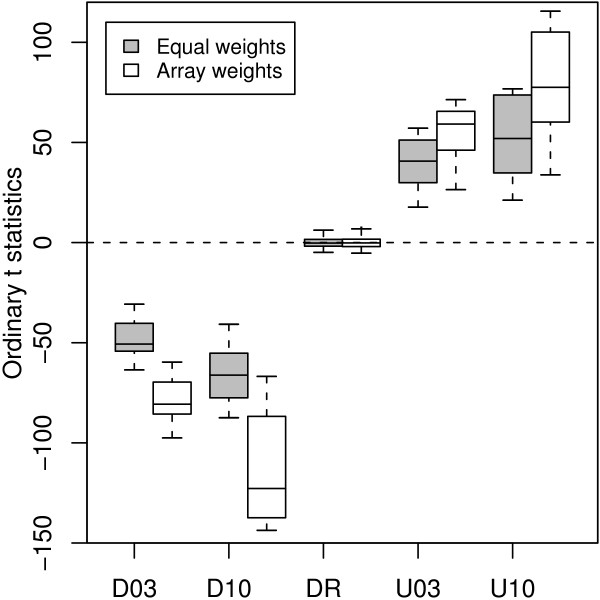
**Ordinary *t*-statistics for the QC LMS controls**. The *t*-statistics were calculated using either equal weights or the array weights (*v*_*j*_) from Figure 4(a). Using array weights in the analysis results in more extreme *t*-statistics for the known differentially expressed controls (D03, D10, U03, U10) which represents a gain in power.

The differential expression status of the LMS control spots are known, so we can use them to assess our ability to distinguish probes which are differentially expressed from those which are not. Figure 6 plots *t*-statistics for testing differential expression for the 120 LMS controls. Ordinary *t*-statistics were calculated using either equal weights or using the array weights shown in Figure 4(a). The *t*-statistics for all classes of ratio controls (D03, D10, U03 and U10) move further from zero when array weights are used while the distribution of *t*-statistics for the dynamic range controls does not noticeably change. This demonstrates that the array quality weights increase statistical power to detect true differential expression without increasing the false discovery rate.

### METH Data

In order to demonstrate our method on a smaller and more complex experiment, we now turn to the METH data. For this experiment, replication takes the form not only of duplicate arrays but also of redundancy between the direct and indirect comparisons available for each pair of treatments. The linear model requires three coefficients to represent differences between the three RNA treatments and the common reference leaving seven residual degrees of freedom. Of primary interest are the coefficients *β*_1-0 _and *β*_3-0 _which measure the gene expression differences 1mM-0mM and 3mM-0mM respectively. The design matrix was generated automatically using the limma software package. The linear model was fitted to all genes in the 10.5 K library and control probes were excluded.

The experimenters who conducted the METH experiment were suspicious of the reliability of the first 4 arrays hybridised, which they believed were not giving consistent results with the last 6 arrays.

Figure 4(b) shows the array weights estimated from this data. Arrays 1 and 4 were assigned the lowest weights of 0.29 and 0.36 respectively. Diagnostic plots of the data [20] reveal that arrays 1 and 4 have high levels of background fluorescence in both channels, which does indeed indicate that these arrays are of poorer quality. The diagnostics do not identify a particular subset of problem spots which could be filtered out, so spot quality methods do not offer a solution. The usual method of dealing with this problem would involve removing these two suspect arrays from further analysis. We now consider the alternative of retaining these microarrays but down-weighting their expression values using empirical array weights. Differential expression was assessed for both methods using moderated *t*-statistics [22] adjusted for multiple testing using the false discovery rate method [33]. Table 3 shows the number of genes for the 1mM-0mM and 3mM-0mM treatment comparisons with adjusted *p*-values (*q*-values) less than 0.05. For the 1mM-0mM comparison, which has two poor quality arrays directly comparing these RNA sources, removal of the worst arrays throws away most of the information on this comparison and results in no differentially expressed genes. Using array weights gives 654 candidate differentially expressed genes for this comparison. Of these genes, 413 are also differentially expressed in the 3mM-0mM comparison and 237 show a monotonic response to dose with the 3mM-0mM fold-change being larger and in the same direction as the 1mM-0mM change. This suggests that many of these genes are worthy candidates for further validation.

## Methods

### Need for new algorithms

We now turn to the problem of computing REML estimates for the array variance parameters in the probe-array variance model (Equation 5). Algorithms for fitting heteroscedastic linear models are already available [34], however the high dimensionality of microarray data limits the usability of conventional algorithms. There are *G + J *- 1 parameters in the variance model and a further *GK *parameters in the linear model itself. The fact that the array parameters in the variance model are shared by all the genes means that the usual strategy of fitting models separately for each gene is not available. Even computers with many gigabytes of memory will run into memory limits using conventional algorithms with *G *much larger than around 50. Using a conventional algorithm for a typical microarray experiment with tens of thousands of genes is out of the question.

The basic difficulty from an algorithmic point of view is not the large number of expression values but rather the large number of parameters to be estimated. In the next section we develop a strategy for eliminating the gene-wise parameters ***β***_*g *_and *δ*_*g *_from the estimation problem.

### Nested iterations

Conditional on the array variance factors *γ*_*j*_, the gene-wise coefficients ***β***_*g *_and variances *δ*_*g *_can be computed in closed form using weighted least squares as described in the linear models section (Equation 3). The method of *nested iterations *is a strategy to reduce the dimension of an estimation problem by eliminating conditionally estimable parameters [35]. The idea is applied here to eliminate the gene-specific parameters from the REML likelihood function. This reduces the estimation problem to one involving just the *J *- 1 array weights.

Explicit expressions for the REML log-likelihood for heteroscedastic models such as ours can be found in [24] and [34]. Write *f*(**y**_*g*_; *δ*_*g*_, **γ**) for the contribution to the REML log-likelihood for gene *g *with **γ **= (*γ*_1_, ..., *γ*_*J*-1_)^*T*^. The REML likelihood already has the property that the linear model parameters ***β***_*g *_are eliminated. The REML log-likelihood to be maximised is

ℓ(**y**_1_, ..., **y**_*G*_; *δ*_1_, ..., *δ *_*G*_, **γ**) = ∑g=1Gf(yg;δg,γ)
 MathType@MTEF@5@5@+=feaafiart1ev1aaatCvAUfKttLearuWrP9MDH5MBPbIqV92AaeXatLxBI9gBaebbnrfifHhDYfgasaacH8akY=wiFfYdH8Gipec8Eeeu0xXdbba9frFj0=OqFfea0dXdd9vqai=hGuQ8kuc9pgc9s8qqaq=dirpe0xb9q8qiLsFr0=vr0=vr0dc8meaabaqaciaacaGaaeqabaqabeGadaaakeaadaaeWbqaaiabdAgaMjabcIcaOGqabiab=Lha5naaBaaaleaacqWGNbWzaeqaaOGaei4oaSdcciGae4hTdq2aaSbaaSqaaiabdEgaNbqabaGccqGGSaaliiqacqqFZoWzcqGGPaqkaSqaaiabdEgaNjabg2da9iabigdaXaqaaiabdEeahbqdcqGHris5aaaa@4028@     (6)

Rather than deal with this large dimensional problem, we eliminate the *δ*_*g *_by considering the profile REML likelihood for **γ**. Write δ^g|γ
 MathType@MTEF@5@5@+=feaafiart1ev1aaatCvAUfKttLearuWrP9MDH5MBPbIqV92AaeXatLxBI9gBaebbnrfifHhDYfgasaacH8akY=wiFfYdH8Gipec8Eeeu0xXdbba9frFj0=OqFfea0dXdd9vqai=hGuQ8kuc9pgc9s8qqaq=dirpe0xb9q8qiLsFr0=vr0=vr0dc8meaabaqaciaacaGaaeqabaqabeGadaaakeaaiiGacuWF0oazgaqcamaaBaaaleaacqWGNbWzcqGG8baFcqWFZoWzaeqaaaaa@330D@ for the value of *δ*_*g *_which maximises *f*(**y**_*g*_; *δ*_*g*_, **γ**) for given **γ**. The profile REML log-likelihood for **γ **is

ℓ_*p*_(**y**_1_, ..., **y**_*G*_; **γ**) = ∑g=1Gf(yg;δ^g|γ,γ)
 MathType@MTEF@5@5@+=feaafiart1ev1aaatCvAUfKttLearuWrP9MDH5MBPbIqV92AaeXatLxBI9gBaebbnrfifHhDYfgasaacH8akY=wiFfYdH8Gipec8Eeeu0xXdbba9frFj0=OqFfea0dXdd9vqai=hGuQ8kuc9pgc9s8qqaq=dirpe0xb9q8qiLsFr0=vr0=vr0dc8meaabaqaciaacaGaaeqabaqabeGadaaakeaadaaeWbqaaiabdAgaMjabcIcaOGqabiab=Lha5naaBaaaleaacqWGNbWzaeqaaOGaei4oaSdcciGaf4hTdqMbaKaadaWgaaWcbaGaem4zaCMaeiiFaWNae43SdCgabeaakiabcYcaSGGabiab9n7aNjabcMcaPaWcbaGaem4zaCMaeyypa0JaeGymaedabaGaem4raCeaniabggHiLdaaaa@4359@     (7)

We consider now the nested iteration for maximising the profile likelihood. Write

Ug,γ=∂f(yg;δg,γ)∂γ.     (8)
 MathType@MTEF@5@5@+=feaafiart1ev1aaatCvAUfKttLearuWrP9MDH5MBPbIqV92AaeXatLxBI9gBaebbnrfifHhDYfgasaacH8akY=wiFfYdH8Gipec8Eeeu0xXdbba9frFj0=OqFfea0dXdd9vqai=hGuQ8kuc9pgc9s8qqaq=dirpe0xb9q8qiLsFr0=vr0=vr0dc8meaabaqaciaacaGaaeqabaqabeGadaaakeaacqWGvbqvdaWgaaWcbaGaem4zaCMaeiilaWccciGae83SdCgabeaakiabg2da9maalaaabaGae8NaIyRaemOzayMaeiikaGccbeGae4xEaK3aaSbaaSqaaiabdEgaNbqabaGccqGG7aWocqWF0oazdaWgaaWcbaGaem4zaCgabeaakiabcYcaSGGabiab9n7aNjabcMcaPaqaaiab=jGi2kab9n7aNbaacqGGUaGlcaWLjaGaaCzcamaabmaabaGaeGioaGdacaGLOaGaayzkaaaaaa@48E7@

Also let

Ag=(Ag,δδAg,δγAg,γδAg,γγ)     (9)
 MathType@MTEF@5@5@+=feaafiart1ev1aaatCvAUfKttLearuWrP9MDH5MBPbIqV92AaeXatLxBI9gBaebbnrfifHhDYfgasaacH8akY=wiFfYdH8Gipec8Eeeu0xXdbba9frFj0=OqFfea0dXdd9vqai=hGuQ8kuc9pgc9s8qqaq=dirpe0xb9q8qiLsFr0=vr0=vr0dc8meaabaqaciaacaGaaeqabaqabeGadaaakeaacqWGbbqqdaWgaaWcbaGaem4zaCgabeaakiabg2da9maabmaabaqbaeqabiGaaaqaaiabdgeabnaaBaaaleaacqWGNbWzcqGGSaaliiGacqWF0oazcqWF0oazaeqaaaGcbaGaemyqae0aaSbaaSqaaiabdEgaNjabcYcaSiab=r7aKjab=n7aNbqabaaakeaacqWGbbqqdaWgaaWcbaGaem4zaCMaeiilaWIae83SdCMae8hTdqgabeaaaOqaaiabdgeabnaaBaaaleaacqWGNbWzcqGGSaalcqWFZoWzcqWFZoWzaeqaaaaaaOGaayjkaiaawMcaaiaaxMaacaWLjaWaaeWaaeaacqaI5aqoaiaawIcacaGLPaaaaaa@50A4@

be the REML information matrix for gene *g*. The derivative of *f*(**y**_*g*_; δ^g|γ
 MathType@MTEF@5@5@+=feaafiart1ev1aaatCvAUfKttLearuWrP9MDH5MBPbIqV92AaeXatLxBI9gBaebbnrfifHhDYfgasaacH8akY=wiFfYdH8Gipec8Eeeu0xXdbba9frFj0=OqFfea0dXdd9vqai=hGuQ8kuc9pgc9s8qqaq=dirpe0xb9q8qiLsFr0=vr0=vr0dc8meaabaqaciaacaGaaeqabaqabeGadaaakeaaiiGacuWF0oazgaqcamaaBaaaleaacqWGNbWzcqGG8baFcqWFZoWzaeqaaaaa@330D@, **γ**) with respect to **γ **is simply *U*_*g*,*γ *_evaluated at *δ*_*g *_= δ^g|γ
 MathType@MTEF@5@5@+=feaafiart1ev1aaatCvAUfKttLearuWrP9MDH5MBPbIqV92AaeXatLxBI9gBaebbnrfifHhDYfgasaacH8akY=wiFfYdH8Gipec8Eeeu0xXdbba9frFj0=OqFfea0dXdd9vqai=hGuQ8kuc9pgc9s8qqaq=dirpe0xb9q8qiLsFr0=vr0=vr0dc8meaabaqaciaacaGaaeqabaqabeGadaaakeaaiiGacuWF0oazgaqcamaaBaaaleaacqWGNbWzcqGG8baFcqWFZoWzaeqaaaaa@330D@. The information matrix for **γ** from gene *g*, conditional on *δ*_*g *_= δ^g|γ
 MathType@MTEF@5@5@+=feaafiart1ev1aaatCvAUfKttLearuWrP9MDH5MBPbIqV92AaeXatLxBI9gBaebbnrfifHhDYfgasaacH8akY=wiFfYdH8Gipec8Eeeu0xXdbba9frFj0=OqFfea0dXdd9vqai=hGuQ8kuc9pgc9s8qqaq=dirpe0xb9q8qiLsFr0=vr0=vr0dc8meaabaqaciaacaGaaeqabaqabeGadaaakeaaiiGacuWF0oazgaqcamaaBaaaleaacqWGNbWzcqGG8baFcqWFZoWzaeqaaaaa@330D@ is

Ag,γ⋅δ=Ag,γγ−Ag,γδAg,δδ−1Ag,δγ     (10)
 MathType@MTEF@5@5@+=feaafiart1ev1aaatCvAUfKttLearuWrP9MDH5MBPbIqV92AaeXatLxBI9gBaebbnrfifHhDYfgasaacH8akY=wiFfYdH8Gipec8Eeeu0xXdbba9frFj0=OqFfea0dXdd9vqai=hGuQ8kuc9pgc9s8qqaq=dirpe0xb9q8qiLsFr0=vr0=vr0dc8meaabaqaciaacaGaaeqabaqabeGadaaakeaacqWGbbqqdaWgaaWcbaGaem4zaCMaeiilaWccciGae83SdCMaeyyXICTae8hTdqgabeaakiabg2da9iabdgeabnaaBaaaleaacqWGNbWzcqGGSaalcqWFZoWzcqWFZoWzaeqaaOGaeyOeI0Iaemyqae0aaSbaaSqaaiabdEgaNjabcYcaSiab=n7aNjab=r7aKbqabaGccqWGbbqqdaqhaaWcbaGaem4zaCMaeiilaWIae8hTdqMae8hTdqgabaGaeyOeI0IaeGymaedaaOGaemyqae0aaSbaaSqaaiabdEgaNjabcYcaSiab=r7aKjab=n7aNbqabaGccaWLjaGaaCzcamaabmaabaGaeGymaeJaeGimaadacaGLOaGaayzkaaaaaa@5920@

evaluated at *δ*_*g *_= δ^g|γ
 MathType@MTEF@5@5@+=feaafiart1ev1aaatCvAUfKttLearuWrP9MDH5MBPbIqV92AaeXatLxBI9gBaebbnrfifHhDYfgasaacH8akY=wiFfYdH8Gipec8Eeeu0xXdbba9frFj0=OqFfea0dXdd9vqai=hGuQ8kuc9pgc9s8qqaq=dirpe0xb9q8qiLsFr0=vr0=vr0dc8meaabaqaciaacaGaaeqabaqabeGadaaakeaaiiGacuWF0oazgaqcamaaBaaaleaacqWGNbWzcqGG8baFcqWFZoWzaeqaaaaa@330D@[35].

The derivative of the profile REML log-likelihood ℓ_*p *_therefore is

Uγ=∑g=1GUg,γ     (11)
 MathType@MTEF@5@5@+=feaafiart1ev1aaatCvAUfKttLearuWrP9MDH5MBPbIqV92AaeXatLxBI9gBaebbnrfifHhDYfgasaacH8akY=wiFfYdH8Gipec8Eeeu0xXdbba9frFj0=OqFfea0dXdd9vqai=hGuQ8kuc9pgc9s8qqaq=dirpe0xb9q8qiLsFr0=vr0=vr0dc8meaabaqaciaacaGaaeqabaqabeGadaaakeaacqWGvbqvdaWgaaWcbaacciGae83SdCgabeaakiabg2da9maaqahabaGaemyvau1aaSbaaSqaaiabdEgaNjabcYcaSiab=n7aNbqabaaabaGaem4zaCMaeyypa0JaeGymaedabaGaem4raCeaniabggHiLdGccaWLjaGaaCzcamaabmaabaGaeGymaeJaeGymaedacaGLOaGaayzkaaaaaa@4153@

and the information matrix associated with ℓ_*p *_is

Aγ⋅δ=∑g=1GAg,γ⋅δ     (12)
 MathType@MTEF@5@5@+=feaafiart1ev1aaatCvAUfKttLearuWrP9MDH5MBPbIqV92AaeXatLxBI9gBaebbnrfifHhDYfgasaacH8akY=wiFfYdH8Gipec8Eeeu0xXdbba9frFj0=OqFfea0dXdd9vqai=hGuQ8kuc9pgc9s8qqaq=dirpe0xb9q8qiLsFr0=vr0=vr0dc8meaabaqaciaacaGaaeqabaqabeGadaaakeaacqWGbbqqdaWgaaWcbaacciGae83SdCMaeyyXICTae8hTdqgabeaakiabg2da9maaqahabaGaemyqae0aaSbaaSqaaiabdEgaNjabcYcaSiab=n7aNjabgwSixlab=r7aKbqabaaabaGaem4zaCMaeyypa0JaeGymaedabaGaem4raCeaniabggHiLdGccaWLjaGaaCzcamaabmaabaGaeGymaeJaeGOmaidacaGLOaGaayzkaaaaaa@48D9@

evaluated at *δ*_*g *_= δ^g|γ
 MathType@MTEF@5@5@+=feaafiart1ev1aaatCvAUfKttLearuWrP9MDH5MBPbIqV92AaeXatLxBI9gBaebbnrfifHhDYfgasaacH8akY=wiFfYdH8Gipec8Eeeu0xXdbba9frFj0=OqFfea0dXdd9vqai=hGuQ8kuc9pgc9s8qqaq=dirpe0xb9q8qiLsFr0=vr0=vr0dc8meaabaqaciaacaGaaeqabaqabeGadaaakeaaiiGacuWF0oazgaqcamaaBaaaleaacqWGNbWzcqGG8baFcqWFZoWzaeqaaaaa@330D@. The REML estimate of **γ **can be evaluated by the nested scoring iteration

**γ**^(*i*+1) ^= **γ**^(*i*) ^+ Aγ⋅δ−1
 MathType@MTEF@5@5@+=feaafiart1ev1aaatCvAUfKttLearuWrP9MDH5MBPbIqV92AaeXatLxBI9gBaebbnrfifHhDYfgasaacH8akY=wiFfYdH8Gipec8Eeeu0xXdbba9frFj0=OqFfea0dXdd9vqai=hGuQ8kuc9pgc9s8qqaq=dirpe0xb9q8qiLsFr0=vr0=vr0dc8meaabaqaciaacaGaaeqabaqabeGadaaakeaacqWGbbqqdaqhaaWcbaacciGae83SdCMaeyyXICTae8hTdqgabaGaeyOeI0IaeGymaedaaaaa@3559@*U*_*γ *_    (13)

where **γ**^(*i*) ^is the *i*th iterated value and *A*_*γ*·*δ *_and *U*_*γ *_are to be evaluated at **γ **= **γ**^(*i*)^. The iteration will begin from a suitable starting value **γ**^(0)^.

### Full scoring iterations

In this section, convenient expressions will be derived for the quantities *A*_*γ*·*δ *_and *U*_*γ*_. For any value of **γ**, the least squares estimator for ***β***_*g *_can be computed using weighted least squares computations (Equation 3) with working weights wgj∗
 MathType@MTEF@5@5@+=feaafiart1ev1aaatCvAUfKttLearuWrP9MDH5MBPbIqV92AaeXatLxBI9gBaebbnrfifHhDYfgasaacH8akY=wiFfYdH8Gipec8Eeeu0xXdbba9frFj0=OqFfea0dXdd9vqai=hGuQ8kuc9pgc9s8qqaq=dirpe0xb9q8qiLsFr0=vr0=vr0dc8meaabaqaciaacaGaaeqabaqabeGadaaakeaacqWG3bWDdaqhaaWcbaGaem4zaCMaemOAaOgabaGaey4fIOcaaaaa@31F3@ replacing the prior weights *w*_*gj*_. The standardised residuals from this regression are

egj=wgj*1/2(ygj−xjTβ^g),     (14)
 MathType@MTEF@5@5@+=feaafiart1ev1aaatCvAUfKttLearuWrP9MDH5MBPbIqV92AaeXatLxBI9gBaebbnrfifHhDYfgasaacH8akY=wiFfYdH8Gipec8Eeeu0xXdbba9frFj0=OqFfea0dXdd9vqai=hGuQ8kuc9pgc9s8qqaq=dirpe0xb9q8qiLsFr0=vr0=vr0dc8meaabaqaciaacaGaaeqabaqabeGadaaakeaacqWGLbqzdaWgaaWcbaGaem4zaCMaemOAaOgabeaakiabg2da9iabdEha3naaDaaaleaacqWGNbWzcqWGQbGAaeaacqGGQaGkcqaIXaqmcqGGVaWlcqaIYaGmaaGccqGGOaakcqWG5bqEdaWgaaWcbaGaem4zaCMaemOAaOgabeaakiabgkHiTGqaaiab=Hha4naaDaaaleaacqWGQbGAaeaacqWGubavaaaccmGccuGFYoGygaqcamaaBaaaleaacqWGNbWzaeqaaOGaeiykaKIaeiilaWIaaCzcaiaaxMaadaqadaqaaiabigdaXiabisda0aGaayjkaiaawMcaaaaa@4E14@

where **x**_*j *_is the *j*th row of *X*.

Let

Hg=Σg−1/2X(XTΣg−1X)−1XTΣg−1/2=(hg,jk)     (15)
 MathType@MTEF@5@5@+=feaafiart1ev1aaatCvAUfKttLearuWrP9MDH5MBPbIqV92AaeXatLxBI9gBaebbnrfifHhDYfgasaacH8akY=wiFfYdH8Gipec8Eeeu0xXdbba9frFj0=OqFfea0dXdd9vqai=hGuQ8kuc9pgc9s8qqaq=dirpe0xb9q8qiLsFr0=vr0=vr0dc8meaabaqaciaacaGaaeqabaqabeGadaaakeaacqWGibasdaWgaaWcbaGaem4zaCgabeaakiabg2da9iabfo6atnaaDaaaleaacqWGNbWzaeaacqGHsislcqaIXaqmcqGGVaWlcqaIYaGmaaGccqWGybawcqGGOaakcqWGybawdaahaaWcbeqaaiabdsfaubaakiabfo6atnaaDaaaleaacqWGNbWzaeaacqGHsislcqaIXaqmaaGccqWGybawcqGGPaqkdaahaaWcbeqaaiabgkHiTiabigdaXaaakiabdIfaynaaCaaaleqabaGaemivaqfaaOGaeu4Odm1aa0baaSqaaiabdEgaNbqaaiabgkHiTiabigdaXiabc+caViabikdaYaaakiabg2da9iabcIcaOiabdIgaOnaaBaaaleaacqWGNbWzcqGGSaalcqWGQbGAcqWGRbWAaeqaaOGaeiykaKIaaCzcaiaaxMaadaqadaqaaiabigdaXiabiwda1aGaayjkaiaawMcaaaaa@5C3E@

be the projection matrix from the regression and write *h*_*gj *_= *h*_*g,jj *_for the diagonal elements or leverages of *H*_*g*_. Finally, let *Z *be the *J *× *J *design matrix

Z=(110⋯0101⋯0⋮⋮⋮⋱⋮100⋯11−1−1⋯−1)     (16)
 MathType@MTEF@5@5@+=feaafiart1ev1aqatCvAUfKttLearuWrP9MDH5MBPbIqV92AaeXatLxBI9gBaebbnrfifHhDYfgasaacH8akY=wiFfYdH8Gipec8Eeeu0xXdbba9frFj0=OqFfea0dXdd9vqai=hGuQ8kuc9pgc9s8qqaq=dirpe0xb9q8qiLsFr0=vr0=vr0dc8meaabaqaciaacaGaaeqabaqabeGadaaakeaacqWGAbGwcqGH9aqpdaqadaqaauaabeaafuaaaaaabaGaeGymaedabaGaeGymaedabaGaeGimaadabaGaeS47IWeabaGaeGimaadabaGaeGymaedabaGaeGimaadabaGaeGymaedabaGaeS47IWeabaGaeGimaadabaGaeSO7I0eabaGaeSO7I0eabaGaeSO7I0eabaGaeSy8I8eabaGaeSO7I0eabaGaeGymaedabaGaeGimaadabaGaeGimaadabaGaeS47IWeabaGaeGymaedabaGaeGymaedabaGaeyOeI0IaeGymaedabaGaeyOeI0IaeGymaedabaGaeS47IWeabaGaeyOeI0IaeGymaedaaaGaayjkaiaawMcaaiaaxMaacaWLjaWaaeWaaeaacqaIXaqmcqaI2aGnaiaawIcacaGLPaaaaaa@5878@

Using these expressions we can write down computable expressions for quantities from the previous section. The conditional REML estimator of *δ*_*g *_is δ^g|γ
 MathType@MTEF@5@5@+=feaafiart1ev1aaatCvAUfKttLearuWrP9MDH5MBPbIqV92AaeXatLxBI9gBaebbnrfifHhDYfgasaacH8akY=wiFfYdH8Gipec8Eeeu0xXdbba9frFj0=OqFfea0dXdd9vqai=hGuQ8kuc9pgc9s8qqaq=dirpe0xb9q8qiLsFr0=vr0=vr0dc8meaabaqaciaacaGaaeqabaqabeGadaaakeaaiiGacuWF0oazgaqcamaaBaaaleaacqWGNbWzcqGG8baFcqWFZoWzaeqaaaaa@330D@ = logsg|γ2
 MathType@MTEF@5@5@+=feaafiart1ev1aaatCvAUfKttLearuWrP9MDH5MBPbIqV92AaeXatLxBI9gBaebbnrfifHhDYfgasaacH8akY=wiFfYdH8Gipec8Eeeu0xXdbba9frFj0=OqFfea0dXdd9vqai=hGuQ8kuc9pgc9s8qqaq=dirpe0xb9q8qiLsFr0=vr0=vr0dc8meaabaqaciaacaGaaeqabaqabeGadaaakeaacqWGZbWCdaqhaaWcbaGaem4zaCMaeiiFaWhcciGae83SdCgabaGaeGOmaidaaaaa@33BF@ with

sg|γ2=1J−K∑j=1Jegj2.     (17)
 MathType@MTEF@5@5@+=feaafiart1ev1aaatCvAUfKttLearuWrP9MDH5MBPbIqV92AaeXatLxBI9gBaebbnrfifHhDYfgasaacH8akY=wiFfYdH8Gipec8Eeeu0xXdbba9frFj0=OqFfea0dXdd9vqai=hGuQ8kuc9pgc9s8qqaq=dirpe0xb9q8qiLsFr0=vr0=vr0dc8meaabaqaciaacaGaaeqabaqabeGadaaakeaacqWGZbWCdaqhaaWcbaGaem4zaCMaeiiFaWhcciGae83SdCgabaGaeGOmaidaaOGaeyypa0ZaaSaaaeaacqaIXaqmaeaacqWGkbGscqGHsislcqWGlbWsaaWaaabCaeaacqWGLbqzdaqhaaWcbaGaem4zaCMaemOAaOgabaGaeGOmaidaaaqaaiabdQgaQjabg2da9iabigdaXaqaaiabdQeakbqdcqGHris5aOGaeiOla4IaaCzcaiaaxMaadaqadaqaaiabigdaXiabiEda3aGaayjkaiaawMcaaaaa@4A6C@

The score vector for *γ *is

Ug,γ=12Z2Tzg     (18)
 MathType@MTEF@5@5@+=feaafiart1ev1aaatCvAUfKttLearuWrP9MDH5MBPbIqV92AaeXatLxBI9gBaebbnrfifHhDYfgasaacH8akY=wiFfYdH8Gipec8Eeeu0xXdbba9frFj0=OqFfea0dXdd9vqai=hGuQ8kuc9pgc9s8qqaq=dirpe0xb9q8qiLsFr0=vr0=vr0dc8meaabaqaciaacaGaaeqabaqabeGadaaakeaacqWGvbqvdaWgaaWcbaGaem4zaCMaeiilaWccciGae83SdCgabeaakiabg2da9maalaaabaGaeGymaedabaGaeGOmaidaaiabdQfaAnaaDaaaleaacqaIYaGmaeaacqWGubavaaacbeGccqGF6bGEdaWgaaWcbaGaem4zaCgabeaakiaaxMaacaWLjaWaaeWaaeaacqaIXaqmcqaI4aaoaiaawIcacaGLPaaaaaa@4053@

where *Z*_2 _is the last *J *- 1 columns of *Z *and **z**_*g *_is the vector with components

zgj=egj2/sg|γ2−(1−hgj)     (19)
 MathType@MTEF@5@5@+=feaafiart1ev1aaatCvAUfKttLearuWrP9MDH5MBPbIqV92AaeXatLxBI9gBaebbnrfifHhDYfgasaacH8akY=wiFfYdH8Gipec8Eeeu0xXdbba9frFj0=OqFfea0dXdd9vqai=hGuQ8kuc9pgc9s8qqaq=dirpe0xb9q8qiLsFr0=vr0=vr0dc8meaabaqaciaacaGaaeqabaqabeGadaaakeaacqWG6bGEdaWgaaWcbaGaem4zaCMaemOAaOgabeaakiabg2da9iabdwgaLnaaDaaaleaacqWGNbWzcqWGQbGAaeaacqaIYaGmaaGccqGGVaWlcqWGZbWCdaqhaaWcbaGaem4zaCMaeiiFaWhcciGae83SdCgabaGaeGOmaidaaOGaeyOeI0IaeiikaGIaeGymaeJaeyOeI0IaemiAaG2aaSbaaSqaaiabdEgaNjabdQgaQbqabaGccqGGPaqkcaWLjaGaaCzcamaabmaabaGaeGymaeJaeGyoaKdacaGLOaGaayzkaaaaaa@4CC8@

for *j *= 1, ..., *J*. The information matrix is

Ag=12ZTVgZ     (20)
 MathType@MTEF@5@5@+=feaafiart1ev1aaatCvAUfKttLearuWrP9MDH5MBPbIqV92AaeXatLxBI9gBaebbnrfifHhDYfgasaacH8akY=wiFfYdH8Gipec8Eeeu0xXdbba9frFj0=OqFfea0dXdd9vqai=hGuQ8kuc9pgc9s8qqaq=dirpe0xb9q8qiLsFr0=vr0=vr0dc8meaabaqaciaacaGaaeqabaqabeGadaaakeaacqWGbbqqdaWgaaWcbaGaem4zaCgabeaakiabg2da9maalaaabaGaeGymaedabaGaeGOmaidaaiabdQfaAnaaCaaaleqabaGaemivaqfaaOGaemOvay1aaSbaaSqaaiabdEgaNbqabaGccqWGAbGwcaWLjaGaaCzcamaabmaabaGaeGOmaiJaeGimaadacaGLOaGaayzkaaaaaa@3D8D@

where *V*_*g *_is the *J *× *J *matrix with diagonal elements (1 - hg,jj2
 MathType@MTEF@5@5@+=feaafiart1ev1aaatCvAUfKttLearuWrP9MDH5MBPbIqV92AaeXatLxBI9gBaebbnrfifHhDYfgasaacH8akY=wiFfYdH8Gipec8Eeeu0xXdbba9frFj0=OqFfea0dXdd9vqai=hGuQ8kuc9pgc9s8qqaq=dirpe0xb9q8qiLsFr0=vr0=vr0dc8meaabaqaciaacaGaaeqabaqabeGadaaakeaacqWGObaAdaqhaaWcbaGaem4zaCMaeiilaWIaemOAaOMaemOAaOgabaGaeGOmaidaaaaa@3415@) and off-diagonal elements hg,jk2
 MathType@MTEF@5@5@+=feaafiart1ev1aaatCvAUfKttLearuWrP9MDH5MBPbIqV92AaeXatLxBI9gBaebbnrfifHhDYfgasaacH8akY=wiFfYdH8Gipec8Eeeu0xXdbba9frFj0=OqFfea0dXdd9vqai=hGuQ8kuc9pgc9s8qqaq=dirpe0xb9q8qiLsFr0=vr0=vr0dc8meaabaqaciaacaGaaeqabaqabeGadaaakeaacqWGObaAdaqhaaWcbaGaem4zaCMaeiilaWIaemOAaOMaem4AaSgabaGaeGOmaidaaaaa@3417@. Efficient algorithms exist to compute *A*_*g *_[34]. Alternatively, it is often satisfactory to approximate the dense matrix *V*_*g *_with the diagonal approximation *V*_*g*1 _= diag(1 - *h*_*g*1_, ..., 1 - *h*_*gJ*_) [25]. With this approximation, a straightforward calculation gives

2*A*_*g*,*γ*·*δ *_= diag(1- **h**_*g*(*J*)_) + (1 - *h*_*gJ*_)*L *- 1J−K
 MathType@MTEF@5@5@+=feaafiart1ev1aaatCvAUfKttLearuWrP9MDH5MBPbIqV92AaeXatLxBI9gBaebbnrfifHhDYfgasaacH8akY=wiFfYdH8Gipec8Eeeu0xXdbba9frFj0=OqFfea0dXdd9vqai=hGuQ8kuc9pgc9s8qqaq=dirpe0xb9q8qiLsFr0=vr0=vr0dc8meaabaqaciaacaGaaeqabaqabeGadaaakeaadaWcaaqaaiabigdaXaqaaiabdQeakjabgkHiTiabdUealbaaaaa@30D5@ (*h*_*gJ *_- **h**_*g*(*J*)_) (*h*_*gJ *_- **h**_*g*(*J*)_)^*T *^    (21)

where **h**_*g*(*J*) _= (*h*_*g*1_, ..., *h*_*gJ *- 1_)^*T *^and *L *is the *J *- 1 × *J *- 1 matrix of 1's. The nested information matrix *A*_*γ*·*δ *_therefore has diagonal elements given by

2Aγ⋅δ,ll=∑g=1G{1−hgl+1−hgJ−(hgJ−hgl)2/(J−K)}     (22)
 MathType@MTEF@5@5@+=feaafiart1ev1aaatCvAUfKttLearuWrP9MDH5MBPbIqV92AaeXatLxBI9gBaebbnrfifHhDYfgasaacH8akY=wiFfYdH8Gipec8Eeeu0xXdbba9frFj0=OqFfea0dXdd9vqai=hGuQ8kuc9pgc9s8qqaq=dirpe0xb9q8qiLsFr0=vr0=vr0dc8meaabaqaciaacaGaaeqabaqabeGadaaakeaacqaIYaGmcqWGbbqqdaWgaaWcbaacciGae83SdCMaeyyXICTae8hTdqMaeiilaWIaemiBaWMaemiBaWgabeaakiabg2da9maaqahabaGaei4EaSNaeGymaeJaeyOeI0IaemiAaG2aaSbaaSqaaiabdEgaNjabdYgaSbqabaGccqGHRaWkcqaIXaqmcqGHsislcqWGObaAdaWgaaWcbaGaem4zaCMaemOsaOeabeaakiabgkHiTiabcIcaOiabdIgaOnaaBaaaleaacqWGNbWzcqWGkbGsaeqaaOGaeyOeI0IaemiAaG2aaSbaaSqaaiabdEgaNjabdYgaSbqabaGccqGGPaqkdaahaaWcbeqaaiabikdaYaaakiabc+caViabcIcaOiabdQeakjabgkHiTiabdUealjabcMcaPiabc2ha9jaaxMaacaWLjaWaaeWaaeaacqaIYaGmcqaIYaGmaiaawIcacaGLPaaaaSqaaiabdEgaNjabg2da9iabigdaXaqaaiabdEeahbqdcqGHris5aaaa@671C@

and off-diagonal elements given by

2Aγ⋅δ,lm=∑g=1G{(1−hgJ)−1J−K(hgJ−hgl)(hgJ−hgm)}     (23)
 MathType@MTEF@5@5@+=feaafiart1ev1aaatCvAUfKttLearuWrP9MDH5MBPbIqV92AaeXatLxBI9gBaebbnrfifHhDYfgasaacH8akY=wiFfYdH8Gipec8Eeeu0xXdbba9frFj0=OqFfea0dXdd9vqai=hGuQ8kuc9pgc9s8qqaq=dirpe0xb9q8qiLsFr0=vr0=vr0dc8meaabaqaciaacaGaaeqabaqabeGadaaakeaacqaIYaGmcqWGbbqqdaWgaaWcbaacciGae83SdCMaeyyXICTae8hTdqMaeiilaWIaemiBaWMaemyBa0gabeaakiabg2da9maaqahabaGaei4EaSNaeiikaGIaeGymaeJaeyOeI0IaemiAaG2aaSbaaSqaaiabdEgaNjabdQeakbqabaGccqGGPaqkcqGHsisldaWcaaqaaiabigdaXaqaaiabdQeakjabgkHiTiabdUealbaacqGGOaakcqWGObaAdaWgaaWcbaGaem4zaCMaemOsaOeabeaakiabgkHiTiabdIgaOnaaBaaaleaacqWGNbWzcqWGSbaBaeqaaOGaeiykaKIaeiikaGIaemiAaG2aaSbaaSqaaiabdEgaNjabdQeakbqabaGccqGHsislcqWGObaAdaWgaaWcbaGaem4zaCMaemyBa0gabeaakiabcMcaPiabc2ha9jaaxMaacaWLjaWaaeWaaeaacqaIYaGmcqaIZaWmaiaawIcacaGLPaaaaSqaaiabdEgaNjabg2da9iabigdaXaqaaiabdEeahbqdcqGHris5aaaa@69F6@

In matrix terms we can write

2*A*_*γ*·*δ *_= diag(*u*_1_, ..., *u*_*J*-1_) + *u*_*J*_*L *-*N*^*T*^*N*/(*J *-*K*)     (24)

where *N *is the matrix with *i*th row *h*_*gJ*_- **h**_*g*(*J*) _and *u*_*j *_= ∑g=1G(1−hgj)
 MathType@MTEF@5@5@+=feaafiart1ev1aaatCvAUfKttLearuWrP9MDH5MBPbIqV92AaeXatLxBI9gBaebbnrfifHhDYfgasaacH8akY=wiFfYdH8Gipec8Eeeu0xXdbba9frFj0=OqFfea0dXdd9vqai=hGuQ8kuc9pgc9s8qqaq=dirpe0xb9q8qiLsFr0=vr0=vr0dc8meaabaqaciaacaGaaeqabaqabeGadaaakeaadaaeWaqaaiabcIcaOiabigdaXiabgkHiTiabdIgaOnaaBaaaleaacqWGNbWzcqWGQbGAaeqaaOGaeiykaKcaleaacqWGNbWzcqGH9aqpcqaIXaqmaeaacqWGhbWra0GaeyyeIuoaaaa@3AE4@(1 - *h*_*gj*_). With these quantities, the nested scoring iteration (Equation 13) is very memory efficient and can be carried out easily on a standard personal computer.

### Gene-by-gene scoring iterations

Although memory efficient, the nested scoring iteration may still require a lot of computation for large *G *since *G *gene-wise regressions must be evaluated for every iteration. If the prior spot weights are equal, *w*_*gj *_= 1, the gene-wise regressions can be computed very quickly but, if not, a full set of least squares computations must be repeated for each gene and each iteration. In this section we explore a much lighter computation scheme in which only one pass is done through the genes and the array variance parameters are updated for each gene. This results in a very efficient gene-by-gene update algorithm which produces approximate REML estimators for the array weights.

The gene-by-gene update algorithm is given by

**γ**^(*g*+1) ^= **γ**^(*g*) ^+ (Ag,γ⋅δ*
 MathType@MTEF@5@5@+=feaafiart1ev1aaatCvAUfKttLearuWrP9MDH5MBPbIqV92AaeXatLxBI9gBaebbnrfifHhDYfgasaacH8akY=wiFfYdH8Gipec8Eeeu0xXdbba9frFj0=OqFfea0dXdd9vqai=hGuQ8kuc9pgc9s8qqaq=dirpe0xb9q8qiLsFr0=vr0=vr0dc8meaabaqaciaacaGaaeqabaqabeGadaaakeaacqWGbbqqdaqhaaWcbaGaem4zaCMaeiilaWccciGae83SdCMaeyyXICTae8hTdqgabaGaeiOkaOcaaaaa@368F@)^-1 ^*U*_*g*,*γ *_    (25)

where *U*_*g*,*γ *_is as above (Equation 18) while A* is an accumulating information matrix defined by

Ag,γ⋅δ*
 MathType@MTEF@5@5@+=feaafiart1ev1aaatCvAUfKttLearuWrP9MDH5MBPbIqV92AaeXatLxBI9gBaebbnrfifHhDYfgasaacH8akY=wiFfYdH8Gipec8Eeeu0xXdbba9frFj0=OqFfea0dXdd9vqai=hGuQ8kuc9pgc9s8qqaq=dirpe0xb9q8qiLsFr0=vr0=vr0dc8meaabaqaciaacaGaaeqabaqabeGadaaakeaacqWGbbqqdaqhaaWcbaGaem4zaCMaeiilaWccciGae83SdCMaeyyXICTae8hTdqgabaGaeiOkaOcaaaaa@368F@ = Ag−1,γ⋅δ*
 MathType@MTEF@5@5@+=feaafiart1ev1aaatCvAUfKttLearuWrP9MDH5MBPbIqV92AaeXatLxBI9gBaebbnrfifHhDYfgasaacH8akY=wiFfYdH8Gipec8Eeeu0xXdbba9frFj0=OqFfea0dXdd9vqai=hGuQ8kuc9pgc9s8qqaq=dirpe0xb9q8qiLsFr0=vr0=vr0dc8meaabaqaciaacaGaaeqabaqabeGadaaakeaacqWGbbqqdaqhaaWcbaGaem4zaCMaeyOeI0IaeGymaeJaeiilaWccciGae83SdCMaeyyXICTae8hTdqgabaGaeiOkaOcaaaaa@386C@ + *A*_*g*,*γ*·*δ *_    (26)

where *A*_*g*,*γ*·*δ *_is evaluated at **γ**^(*g*) ^and δ^g|γ
 MathType@MTEF@5@5@+=feaafiart1ev1aaatCvAUfKttLearuWrP9MDH5MBPbIqV92AaeXatLxBI9gBaebbnrfifHhDYfgasaacH8akY=wiFfYdH8Gipec8Eeeu0xXdbba9frFj0=OqFfea0dXdd9vqai=hGuQ8kuc9pgc9s8qqaq=dirpe0xb9q8qiLsFr0=vr0=vr0dc8meaabaqaciaacaGaaeqabaqabeGadaaakeaaiiGacuWF0oazgaqcamaaBaaaleaacqWGNbWzcqGG8baFcqWFZoWzaeqaaaaa@330D@. The iteration is started from **γ**^0 ^= **0 **and

A0,γ⋅δ*=10(J−K)JZ2TZ2.     (27)
 MathType@MTEF@5@5@+=feaafiart1ev1aaatCvAUfKttLearuWrP9MDH5MBPbIqV92AaeXatLxBI9gBaebbnrfifHhDYfgasaacH8akY=wiFfYdH8Gipec8Eeeu0xXdbba9frFj0=OqFfea0dXdd9vqai=hGuQ8kuc9pgc9s8qqaq=dirpe0xb9q8qiLsFr0=vr0=vr0dc8meaabaqaciaacaGaaeqabaqabeGadaaakeaacqWGbbqqdaqhaaWcbaGaeGimaaJaeiilaWccciGae83SdCMaeyyXICTae8hTdqgabaGaeiOkaOcaaOGaeyypa0ZaaSaaaeaacqaIXaqmcqaIWaamcqGGOaakcqWGkbGscqGHsislcqWGlbWscqGGPaqkaeaacqWGkbGsaaGaemOwaO1aa0baaSqaaiabikdaYaqaaiabdsfaubaakiabdQfaAnaaBaaaleaacqaIYaGmaeqaaOGaeiOla4IaaCzcaiaaxMaadaqadaqaaiabikdaYiabiEda3aGaayjkaiaawMcaaaaa@4AB7@

These starting values begin the iteration from equal array variances with the information weight of ten genes. The effect of accumulating the information matrix in this way is to gradually decrease the step size of the iteration as the iteration passes through all the genes, resulting in a convergent iteration. The final value **γ**^(*G*) ^is taken as the estimate of **γ **and is used to assign array weights. In our implementation in R [36], this algorithm calculates the array variance parameters in less than a second for the QC LMS data and in around 12 seconds for the METH data set on a 2.0 GHz Pentium M computer. The gene-by-gene nature of the algorithm means that minimal RAM is required for these computations.

While the gene-by-gene update algorithm is fast, it provides only an approximation to the REML estimators **γ**, and we need to check the accuracy of this approximation. To do this, expression values (*y*_*gj*_) were simulated from normal distributions for *J *= 10 arrays and *G *= 10000 genes. The array variance parameters (*γ*_*j*_) were equally spaced over the interval [-1, 1]. As already noted, the REML algorithm is invariant with respect to the gene-wise means and variances, so the gene-specific mean and variance parameters were set to zero in our simulations.

**Figure 7:**
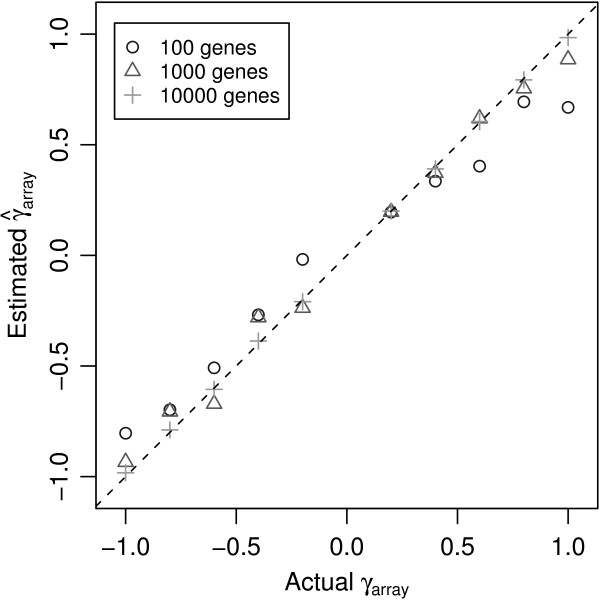
**Estimated versus actual array variance parameters from simulated data**. The gene-by-gene update algorithm was used to estimate the array variance parameters using 100, 1000 and 10000 genes from a simulated data set with 10 arrays. The estimates (γ^j
 MathType@MTEF@5@5@+=feaafiart1ev1aaatCvAUfKttLearuWrP9MDH5MBPbIqV92AaeXatLxBI9gBaebbnrfifHhDYfgasaacH8akY=wiFfYdH8Gipec8Eeeu0xXdbba9frFj0=OqFfea0dXdd9vqai=hGuQ8kuc9pgc9s8qqaq=dirpe0xb9q8qiLsFr0=vr0=vr0dc8meaabaqaciaacaGaaeqabaqabeGadaaakeaaiiGacuWFZoWzgaqcamaaBaaaleaacqWGQbGAaeqaaaaa@2FF3@) are plotted against the true values (*γ*_*j*_). As more genes are included in the iterations the accuracy of the estimates obtained from the gene-by-gene update algorithm improve, although with as few as 100 genes, the values recovered are broadly correct.

Figure 7 shows the estimated versus actual array variance parameters obtained from the gene-by-gene update algorithm after the first 100 genes, first 1000 genes and all 10000 genes respectively. The array variances are broadly correct even after 100 genes and after 1000 genes the accuracy is good. The root mean square errors between the update algorithm estimates and the true values averaged over the ten variance parameters were 0.17, 0.08 and 0.01 after 100, 1000 and 10000 genes respectively. These results indicate that the algorithmic short-cut taken by the gene-by-gene update algorithm does not seriously compromise the accuracy of the array variance estimates.

## Discussion and Conclusion

This article has presented an empirical method for estimating quantitative array quality weights which is integrated into the linear model analysis of microarray data. Computationally efficient algorithms are developed to compute the array quality weights using the well-recognized REML criterion. As well as full REML estimation, a fast gene-by-gene update method which requires only one pass through the genes is described.

Examples of array quality weights which give less influence to the gene expression measurements from unreliable microarrays and relatively more influence to the measurements from reproducible arrays have been presented. In both simulated and real data examples, it has been demonstrated that array weights improve our ability to detect differential expression using standard statistical methods. The graduated approach to array quality has also been shown to be superior to filtering poor quality arrays both in simulations and for an experimental data set. In the simulations, filtering is shown to perform quite poorly, especially in combination with ordinary *t*-statistics. In the data example, filtering resulted in no significant genes to follow up, whereas the weighted analysis provided a few hundred sensible candidates.

The method is restricted for use on data from experiments which include replication with at least two residual degrees of freedom. For simple replicated experiments, a minimum of three arrays are needed and results from simulation studies show that this method is reliable in these situations, even in the presence of non-normally distributed data. Simulations were also used to show that array variance parameters are estimated with greater accuracy when more genes are available for the gene-by-gene update algorithm, and that these computational savings do not seriously compromise the accuracy of the final estimates. As a rule of thumb, we recommend that the full REML array weights be used when there are fewer than 1000 probes and that the gene-by-gene update method be used otherwise. The analysis of the control probes from the QC LMS data set showed that useful array weights can be obtained from the gene-by-gene algorithm with as few as 120 genes. The situation is different when there are no spot weights or missing expression values in the data. In this case the full REML algorithm can be implemented very efficiently and so is recommended for any number of probes.

The empirical array weights form part of the quality and analysis pipeline and are not intended to replace the usual background correction, normalisation and quality assessment steps. In particular, array weights are not designed to account for spot-specific problems. The array weights method is instead designed to incorporate spot quality weights which might arise from gene filtering or from a predictive quality assessment step. The use of zero weights as prior weights *(w*_*gj *_= 0) presents no problems for the method, although some special numerical treatment not discussed here is needed to ensure the sum to zero constraints are satisfied.

The array weight approach is also not intended to replace diagnostic array quality plots such as MA-plots, and arrays which are catastrophically poor quality should still be discarded. Taking a graduated approach to array quality, does however allow arrays of less than ideal quality, which would otherwise have to be discarded, to be kept in the analysis, but down-weighted.

The authors have applied the array weight method to very high quality data sets which featured arrays with low background, well-behaved controls and a good dynamic range of spot intensities. For such data sets, the method assigns approximately equal array weights to each array (data not shown). This indicates that the method does no harm when it is not required.

One further topic that deserves some attention is the use of robust linear models to estimate the gene expression coefficients. The array weights method has the same motivation as robust regression methods, but accumulates information on variability across genes on each array, which gene-wise robust regression methods are unable to do. Another consideration is sample size. While robust methods perform well on large sample problems, many microarray data sets such as the METH experiment consist of a small number of arrays and, in these situations, robust methods may not be suitable.

## Authors' contributions

MER performed the data analyses, coded the algorithms and drafted the manuscript. GKS suggested the model and algorithms, helped with the coding and analyses and finalised the manuscript. The arrays from the QC data set were manufactured and hybridised by DD, RvL and AH. The METH experiment was planned and conducted by JN and AD.

## Figures and Tables

**Table 1:** Summary of QC LMS controls. Theoretical fold-changes for the spike-in control probes in the QC LMS data set are shown. *M *values have been rounded to 2 decimal places.

**Control (abbreviation)**	**Ratio R:G**	***M *= log**_2_**(*R/G*)**
Up-regulated 3-fold (U03)	3:1	log_2 _3 = 1.58
Up-regulated 10-fold (U10)	10:1	log_2 _10 = 3.32
Down-regulated 3-fold (D03)	1:3	-log_2 _3 = -1.58
Down-regulated 10-fold (D10)	1:10	-log_2 _10 = -3.32
Dynamic Range (DR)	1:1	log_2 _1 = 0.00

**Table 2:** Estimates of array weights obtained from 1000 simulated microarray data sets. Means and standard deviations of the array weights estimated from 1000 simulated data sets assuming six different array variance scenarios are shown for normal and non-normal data using the full REML algorithm and the gene-by-gene update algorithm. Accurate estimates with small standard deviations are obtained using the full algorithm. The gene-by-gene update algorithm recovers weights which are generally only slightly flattened towards equal weights.

		**Mean (Standard deviation)**			
**Sim.**	**True weight**	**Normal**		**Non-Normal**	
		Full	Gene-by-gene	Full	Gene-by-gene
1	1.26	1.26 (0.04)	1.23 (0.07)	1.25 (0.04)	1.22 (0.07)
	1.26	1.26 (0.04)	1.23 (0.07)	1.25 (0.04)	1.22 (0.07)
	0.63	0.63 (0.01)	0.66 (0.03)	0.64 (0.01)	0.67 (0.03)
2	2.15	2.16 (0.15)	2.07 (0.14)	2.13 (0.13)	2.04 (0.14)
	2.15	2.16 (0.14)	2.07 (0.13)	2.14 (0.14)	2.04 (0.14)
	0.22	0.22 (0.00)	0.24 (0.01)	0.22 (0.00)	0.24 (0.01)
3	3.68	3.72 (0.33)	2.07 (0.14)	3.54 (0.29)	2.05 (0.15)
	0.74	0.74 (0.04)	1.03 (0.06)	0.75 (0.04)	1.03 (0.06)
	0.37	0.37 (0.02)	0.47 (0.02)	0.38 (0.01)	0.48 (0.02)
4	1.52	1.52 (0.03)	1.50 (0.05)	1.51 (0.03)	1.50 (0.05)
	1.52	1.52 (0.03)	1.50 (0.04)	1.51 (0.03)	1.50 (0.05)
	1.52	1.52 (0.03)	1.50 (0.05)	1.51 (0.03)	1.50 (0.05)
	0.76	0.76 (0.01)	0.77 (0.02)	0.76 (0.01)	0.77 (0.02)
	0.38	0.38 (0.01)	0.38 (0.01)	0.38 (0.01)	0.39 (0.01)
5	2.19	2.19 (0.05)	2.16 (0.07)	2.17 (0.05)	2.14 (0.07)
	2.19	2.19 (0.05)	2.16 (0.07)	2.17 (0.05)	2.14 (0.07)
	2.19	2.19 (0.05)	2.15 (0.07)	2.17 (0.05)	2.14 (0.07)
	0.44	0.44 (0.01)	0.45 (0.01)	0.44 (0.01)	0.45 (0.01)
	0.22	0.22 (0.00)	0.22 (0.00)	0.22 (0.00)	0.23 (0.00)
6	3.44	3.44 (0.12)	3.00 (0.13)	3.37 (0.12)	2.95 (0.13)
	1.72	1.72 (0.04)	1.78 (0.06)	1.72 (0.04)	1.77 (0.06)
	0.86	0.86 (0.02)	0.89 (0.02)	0.86 (0.02)	0.89 (0.02)
	0.57	0.57 (0.01)	0.59 (0.01)	0.58 (0.01)	0.60 (0.01)
	0.34	0.34 (0.01)	0.36 (0.01)	0.35 (0.01)	0.36 (0.01)

**Table 3:** Number of differentially expressed genes for the METH experiment. Counts of candidate differentially expressed genes for the METH experiment obtained after (1) removing arrays with high background levels from the analysis or (2) keeping these arrays in the analysis, but down-weighting their expression values using the array quality weights from Figure 4 (b) are given for each comparison of interest. The false discovery rate was controlled to be less than 0.05 in each case. More candidate genes are discovered using array weights.

**Analysis method**	**Number of genes**	
	**1mM-0mM**	**3mM-0mM**
1. Remove arrays	0	1263
2. Array weights	654	1790
